# Mesencephalic Astrocyte-Derived Neurotrophic Factor (MANF) Is Highly Expressed in Mouse Tissues With Metabolic Function

**DOI:** 10.3389/fendo.2019.00765

**Published:** 2019-11-06

**Authors:** Tatiana Danilova, Emilia Galli, Emmi Pakarinen, Erik Palm, Päivi Lindholm, Mart Saarma, Maria Lindahl

**Affiliations:** HILIFE, Institute of Biotechnology, University of Helsinki, Helsinki, Finland

**Keywords:** MANF, CDNF, ER stress, UPR, pituitary gland, metabolism

## Abstract

Mesencephalic astrocyte-derived neurotrophic factor (MANF) and cerebral dopamine neurotrophic factor (CDNF) form a family of atypical growth factors discovered for their neuroprotective properties in the central nervous system (CNS) in animal models of neurodegenerative diseases. Although their mechanism of protective action still remains unclear, it has been suggested that both MANF and CDNF promote cell survival through regulating the unfolded protein response (UPR), thereby relieving endoplasmic reticulum (ER) stress. Recent studies identified MANF for its emerging roles in metabolic function, inflammation and pancreatic β-cells. We have found that MANF deletion from the pancreas and β-cells leads to postnatal depletion of β-cells and diabetes. Moreover, global MANF-deficiency in mice results in severe diabetes-independent growth retardation. As the expression pattern of MANF in mouse tissues has not been extensively studied, we set out to thoroughly investigate MANF expression in embryonic and adult mice using immunohistochemistry, histochemical X-gal staining, enzyme-linked immunosorbent assay (ELISA), and quantitative reverse transcription PCR (RT-qPCR). We found that MANF is highly expressed in brain neurons regulating energy homeostasis and appetite, as well as in hypothalamic nuclei producing hormones and neuropeptides important for different body functions. Strong expression of MANF was also observed in peripheral mouse tissues and cells with high secretory and metabolic function. These include pituitary gland and interestingly we found that the anterior pituitary gland is smaller in MANF-deficient mice compared to wild-type mice. Consequently, we found reduction in the number of growth hormone- and prolactin-producing cells. This combined with increased expression of UPR genes, reduced number of proliferating cells in the anterior pituitary and dysregulated expression of pituitary hormones might contribute to the severe growth defect seen in the MANF knockout mice. Moreover, in this study we compared MANF and CDNF levels in mouse tissues. Unlike MANF, CDNF protein levels are generally lower in mouse tissues, and the highest levels of CDNF was observed in the tissues with high-energy demands and oxidative roles, including heart, muscle, testis, and brown adipose tissue.

## Introduction

MANF and CDNF are small, two-domain unconventional neurotrophic factors localized to the ER but MANF is also found secreted upon ER stress-induction from cells (1–5).

Both MANF and CDNF are protective and restorative in rodent models of neurodegenerative diseases (6–13). MANF promotes tissue repair and regeneration in the injured retina by modulation of anti-inflammatory response (14). Notably, the trophic effects of MANF apply not only to neurons but also to other cell types. MANF was found to rescue cardiomyocytes from death in a rodent model of heart ischemia (15). MANF delivered by virus vector to the mouse pancreas induced regeneration of β-cells *in vivo* in a Type 1 diabetes (T1D) model and importantly, recombinant human (rh)MANF increased proliferation of both mouse and human β-cells *in vitro* (4, 5, 16). Moreover, rhMANF also protected mouse β-cells from thapsigargin-induced cell death and partially also human β-cells from cytokine-induced cell death (4, 17).

The protective and restorative effects of MANF and CDNF in rodent disease models and in cultured cells *in vitro* have been suggested to depend on their role in modulating UPR in ER stress (18, 19). The ultimate proof for the role of MANF in regulating/dampening ER stress came from our studies in MANF knockout mice showing that insulin-producing β-cells lacking MANF develop severe chronic ER stress leading to decreased β-cell proliferation and cell death (5, 16). ER stress in cells is caused by the accumulation and aggregation of unfolded or misfolded proteins in the ER, which activates the UPR, a signaling cascades attempting to restore the organelle's physiological activity (20). The UPR signaling in mammalian cells is mediated via three major ER transmembrane sensors, protein kinase RNA(PKR)-like ER endoplasmic reticulum kinase (PERK), endoribonuclease inositol-requiring protein 1 (IRE1 α and β) and activating transcription factor 6 (ATF6 α and β) (21). The activation of these sensors leads to the initiation of downstream signaling cascades aiming to restore the normal function of the cell. However, if prolonged and unresolved, UPR leads to cell death (22–24). Inactivation of several UPR genes in mice and humans cause metabolic phenotypes, skeletal, and brain defects (25–30). In addition, dysregulation of the UPR has been suggested to contribute to the pathogenesis of several human disorders, including neurodegenerative diseases, diabetes, metabolic disorders, and autoimmune diseases (31, 32).

Increasing evidence is emerging for the importance of the proper expression level of MANF protein in cells and tissues involved in energy metabolism (33, 34). In addition, data on the circulating levels of MANF in newly diagnosed diabetes patients support roles for MANF in the regulation of systemic metabolic homeostasis (35, 36). MANF-deficiency in mice results in β-cell death and diabetes, and a severe diabetes-independent growth defect (16). In addition, in a clinical exome sequencing screen a homozygote missense mutation in the MANF gene indicative of deficient MANF expression was found in a patient suffering from Type 2 diabetes and obesity, short stature, microcephaly, and other anomalies (37). Reduced MANF expression in β-cells seems to underlie enhanced susceptibility for β-cell apoptosis and diabetes in mice and humans (38).

Our studies and others have demonstrated wide MANF and CDNF expression in both rodent and human brain (5, 6, 13, 33, 39, 40). In peripheral tissues, we have found strong expression of MANF in tissues with a high secretory function such as pancreatic exocrine acinar cells and endocrine islet β-cells, salivary glands, and testicular spermatocytes (5, 16, 39). In addition, the level of MANF protein expression seems to reduce with aging in rodent brain and other metabolic tissues and flies (34, 40). In peripheral mouse tissues, CDNF expression was observed in the adult mouse heart, muscle, salivary glands, testicles, and lungs (6). Hence, it seems that expression patterns of MANF and CDNF partly overlap in the mouse neuronal and non-neuronal tissues.

However, a comprehensive comparison of the expression levels of MANF and CDNF has not been made. In addition, a detailed characterization of MANF expression in metabolic and secretory tissues in mice is lacking. In this study, we have carefully analyzed the expression pattern of MANF in the mouse embryo and juvenile mouse tissues by immunohistochemistry using MANF antibodies validated against *Manf*^−/−^ mouse tissues. In addition, MANF and CDNF mRNA and protein levels in different mouse tissues were compared using and RT-qPCR and in-lab ELISAs, which were constructed and validated for quantitation of mouse MANF and CDNF proteins. We observed that MANF expression is high in mouse tissues and brain areas responsible for metabolic regulation of feeding and energy balance, and in cells of peripheral tissues with high secretory function. The expression of MANF was especially high in several hypothalamic nuclei, the mouse pituitary, thyroid and adrenal glands, all tissues involved in the neuro-endocrine axes important for the regulation of feeding, stress, growth, and development. Consequently, we found that the size of the endocrine adenohypophysis compared to the whole pituitary gland was decreased in the *Manf*^−/−^ mice. The reduced size was consistent with the decreased number of cells producing growth hormone and prolactin, dysregulated expression of pituitary hormones, increased ER stress and apoptosis, all which might contribute to the severe growth defect seen in the *Manf*^−/−^ mice.

## Methods

### Mice and Targeted Embryonic Stem Cells

Mice used in this study were generated from heterozygote breeding of *Manf* ^+/−^ and *Cdnf* ^+/−^ mice (16, 18) (Lindahl et al., submitted manuscript). Tissues from *Manf*^−/−^ and *Cdnf*^−/−^ animals were used as a negative control in immunohistochemistry and ELISA. All mice were maintained on ICR background and housed on a 12-h dark/light cycle, *ad libitum* fed and with free access to water. Finnish Animal Ethics Committee of the State Provincial Office of Southern Finland approved all experimental procedures involving mice.

MANF (Manf_D06, C57BL/6N-*Manf*^*tm*1*a*(*KOMP*)*Wtsi*^) (and CDNF (Cdnf_CO1, C57BL/6N-*Cdnf*^*tm*1*a*(*KOMP*)*Wtsi*^) targeted embryonic stem cell clones were generated by the trans-National Institutes of Health (NIH) Knockout Mouse Project and obtained from the KOMP Repository (http://www.komp.org).

### Islet Isolation

Islet isolation was performed as described previously (16). In brief, islets from 8 weeks old mice were isolated by standard collagenase (Collagenase P; Roche Diagnostic) digestion followed by handpicking under a stereomicroscope.

### RNA Isolation, Reverse Transcription Quantitative PCR (RT-qPCR)

Mouse tissues were collected and fast frozen in liquid nitrogen. RNA isolation, reverse transcription, and RT-qPCR were performed as described previously (16). Total RNA was isolated from mouse tissues or isolated islets by homogenizing them in TriReagent® (Molecular Research Center) according to standard protocol procedures. The RNA amount and purity was measured by NanoDrop ND-1000 spectrophotometer (Thermo Scientific). Glycogen (RNA grade, Thermo Scientific) was used during the RNA precipitation procedure to visualize the pellet. cDNA synthesis was done by reverse transcription reactions with RevertAid™ Premium Reverse Transcriptase (Fermentas UAB, Thermo Fisher Scientific Inc.) according to the manufacturer's protocol. RT-qPCR was performed using LightCycler® 480 SYBR Green I Master (Roche Diagnostics GmbH) and Roche LightCycler® 480 Real-Time PCR System. The expression levels were normalized to the levels of β-actin in the same samples. Primer sequences for *Gh, Prl, Pit1, Tsh*β*, mPomc, Fsh, Lhb, Grp78, Chop, sXbp1, tXbp1, Atf4, Atf6*α*, Atf6*β, *Manf* genes used in this study were from (5, 16, 41). Primer sequences for *Cdnf* gene were F 5′-AGC TGC TCA ACT TTT GCT CA-3′, R 5′-TAG GAT CTT GGT GGC TGC AT-3′.

### Protein Isolation

Mouse tissue samples were homogenized in ice-cold lysis buffer (100 mM Tris-HCl, pH 7.5, 300 mM NaCl, 4 mM EDTA, 0.2% Triton X-100, 2 mM phenylmethylsulfonyl fluoride (PMSF), 1 mM sodium orthovanadate, protease inhibitor cocktail (Complete, Mini EDTA free, Boehringer Mannheim) and incubated on ice for 30 min. Thereafter the homogenates were centrifuged at 13,000 rpm for 30 min at +4°C and supernatants were collected and stored at −80°C for MANF and CDNF ELISAs.

### Mouse MANF ELISA

The concentrations of endogenous MANF in mouse tissue lysates were analyzed by in-lab developed sandwich ELISA. The development and Validation of the mouse (m)MANF ELISA was described in detail elsewhere (42). The sensitivity of mMANF ELISA is 29 pg/ml, and the dynamic range is from 62.5 to 1 000 pg/ml. The ELISA gave no signal from MANF knockout mouse samples, indicating that it was specific for MANF. Briefly, a 96-well plate (MaxiSorp, Nunc) was coated with goat antibodies to MANF (AF3748, R&D^+^ systems) in carbonate coating buffer (pH 9.6) for overnight at 4°C. Next day, after washing with PBS-0.05% Tween (PBST), the wells were blocked with 1% casein in PBST to prevent unspecific binding. Mouse tissue samples and recombinant MANF standard were diluted in blocking buffer and incubated on the plate for overnight at +4°C. On the third day, the wells were washed with PBST, and rabbit anti-MANF antibody (LS-B2688, LSBio) was diluted in blocking buffer and incubated in the wells for 3 h at 37°C. For the detection of bound MANF, a secondary antibody conjugated to horseradish peroxidase (HRP; NA9340V, GE Healthcare) was applied to the plate and incubated for 2 h at room temperature. HRP signal was detected with 3,3′,5,5′-tetramethylbenzidine substrate (DuoSet ELISA Development System, R&D Systems) and absorbance measurement using Victor^3^ reader (Perkin Elmer) at 450 nm and 540 nm (for wavelength correction).

### Mouse CDNF ELISA

The levels of endogenous mouse CDNF in mouse tissue lysates were analyzed by in-lab developed and validated sandwich ELISA. MaxiSorp (Nunc, Fisher Scientific) 96-well microtiter plates were coated overnight at +4°C with 1 μg/ml goat anti-mCDNF (AF5187, R&D Systems) in 0.05 M carbonate/bicarbonate coating buffer (pH 9.6). The next day, unspecific binding to plates was blocked with 3% BSA in PBS for 2 h at room temperature. After blocking, the study and standard (recombinant mCDNF protein, 5187-CD, R&D Systems) samples were diluted in the blocking buffer and applied on the plate as duplicate measurements. The samples were incubated on the plate overnight at +4°C in 100 rpm agitation.

Following day, a detection antibody rabbit anti-CDNF (6) was applied at 0.2 μg/ml in blocking buffer, and the plate was incubated for 3 h at +37°C in 100 rpm agitation. Finally, horse radish peroxidase (HRP)-linked donkey anti-rabbit (GE Healthcare) secondary antibody was diluted at 1:2,000 in blocking buffer and incubated in the plate for 2 h at room temperature in 100 rpm agitation. Before each step, liquids from the previous step were removed, and the wells were washed with PBS-Tween 20 (0.05%). Blocking and washing were performed in a volume of 200 μl, while antibodies and samples were added at 100 μl volume. The bound HRP-linked antibody was visualized by the addition of 3,3′,5,5′-tetramethylbenzidine (DuoSet ELISA Development System, R&D Systems), and absorbance was read at 450 and 540 nm (Victor^3^ reader, Perkin Elmer). MANF and CDNF concentrations in tissue lysates were normalized to the total amount of sample protein, which was analyzed by a DC Protein Assay (Bio-Rad).

Mouse CDNF ELISA was validated for specificity, sensitivity, dynamic range and precision. Specificity of the assays was determined by the response to *Cdnf* ^−/−^ tissue lysates. Sensitivity was determined by the mean absorbance value of ten blank samples added by 3 standard deviations and calculating resulting concentration from the standard curve. The dynamic range of the assay was determined by the accuracy of the back-calculated concentration values for each standard curve point from six individual assays. Within the assay dynamic range, the individual back-calculated accuracy values were within ±10% relative error (% RE = derived concentration/expected concentration × 100%) and precision max 10% coefficient of variation (% CV = SD/mean × 100%). Within-run precision (i.e., intra-assay variation) was determined by running three samples of varying concentration in replicates of ten on different parts of the same microtiter plate. Between-run precision (i.e., inter-assay variation) of the assays was determined by running three samples in duplicate on six independent assay runs performed on different days. Dilutional linearity and recovery of spiked analyte were analyzed in the study samples.

The sensitivity of the mouse CDNF ELISA was 6 pg/ml and the dynamic range from 15.6 to 500 pg/ml (Supplementary Table 1). Intra- and interassay precision was 10.3% CV (9.0–12.1% CV) and 6.8% CV (4.2–8.7% CV), respectively (Supplementary Table 2). The assay gave values below the ELISA sensitivity level for *Cdnf*^−/−^ tissue samples (Supplementary Table 3). The linearity of dilution was within 86–106% RE (*n* = 5, three sequential dilutions per sample, Supplementary Table 4) and recovery of 100 and 250 pg/ml spikes of recombinant mouse CDNF was within 85–135% RE (*n* = 3 per spike, Supplementary Table 5).

### Histological Analysis

Embryonic stem cells were cultured on 0.1% gelatin-coated dishes in ES cell medium (Knockout DMEM™ high glucose, Gibco, 10829–018, 15% FBS, 2 mM GlutaMax™-I (Gibco), 1 mM Non-Essential Amino Acids, 1,000 U/ml LIF and 1 μM β-mercaptoethanol. Cells were fixed in 2% PFA for 1 h, washed several times with PBS and stained with LacZ staining as described below. The induction of the *Manf* gene by reporter gene β-galactosidase expression was analyzed by LacZ staining of embryonic stem cells and whole mount staining of embryonic day (E)7.5 decidua and E9.5 embryos. Immunohistochemistry (IHC) was applied for sectioned E9.5 and E13.5 embryos, and adult mouse tissues.

LacZ staining on whole mount embryos was performed on deciduae containing E7.5 embryos using X-gal solution. First, the removed deciduae were fixed in 2% PFA for 1 h on ice and washed several times in PBS at RT for 2 h. Thereafter deciduae were incubated in staining solution [PBS supplemented with 2 mM MgCl_2_, 5 mM potassium ferrocyanide (K_4_Fe(CN)6-3H_2_0], 5 mM potassium ferricyanide [K3Fe(CN)6, 0.01% NP-40, 0.01% Sodium deoxycholate] containing X-Gal reagent (5-Bromo-4-chloro-3-indolyl beta-D-galactopyranoside) at final concentration of 1 mg/ml for several hours at 37°C in dark. After staining, deciduae were dehydrated and embedded in paraffin followed by sectioning of the samples and counterstaining with nuclear fast red.

For IHC the animals were euthanized by carbon dioxide followed by cervical dislocation. Mouse tissues or embryos were collected and fixed in 4% paraformaldehyde (PFA) for 72 h following embedding to paraffin. Deparaffinised 5 μm thick sections were boiled in citrate buffer pH 6.0 for retrieval of the antigen, rinsed in Tris-buffered saline (TBS) (pH 7.4) and kept in 0.3% H_2_O_2_ solution for 30 min to suppress endogenous peroxidase activity. Next, the sections were washed in TBS with 0.1% Tween (TBST) and blocked with 5% goat or 5% donkey serum for 1–2 h at room temperature. Samples were incubated with primary antibodies (Table 1) in the appropriate blocking buffer overnight at 4°C. For light microscopy peroxidase staining, sections were incubated with biotinylated secondary antibodies for 1 h at room temperature, washed in TBST and incubated with avidin-biotinylated enzyme complex (ABC) according to manufacturer's instructions. Finally, after the washing procedure in TBST, samples were developed with 3, 3'-Diaminobenzidine (DAB), counterstained with hematoxylin and mounted with Depex. For immunofluorescence staining, sections were subjected to antigen retrieval and incubated with primary antibody o/n, followed by incubation with appropriate Alexa-conjugated secondary antibodies, for 4 h at room temperature, washed in TBST and mounted with mounting medium containing DAPI (Vectashield, Vector Laboratories, Burlingame, CA, USA) to visualize nuclear staining.

**Table 1:** Primary antibodies used in immunohistochemical studies.

**Antigen**	**Catolog number**	**Host**	**Dilution**	**Source**
MANF	310–100, batch A12ManfPab-005, 0.45 mg/ml	Rabbit	1:300	Icosagen, Estonia
ARP (MANF) C-19	sc-34560	Goat	1:500	Santa Cruz
MANF C terminal	LS-B2688	Rabbit	1:2000	LSBio
NeuN	MAB377	Mouse	1:200	Chemicon
Vasopressin	AB1565	Rabbit	1:1000	Millipore
Oxytocin	AB911	Rabbit	1:1000	Millipore
ChAT	AB144P	Goat	1:200	Millipore
GFAP	G3893	Mouse	1:500	Sigma
TH	MAB318	Mouse	1:200	Millipore
Calbindin D28K (C-20)	sc7691	Goat	1:500	Santa Cruz
PGP 9.5	ab10410	Gunia pig	1:500	Abcam
Calcitonin	A0576	Rabbit	1:200	Dako, Denmark
GH	AFP-5672099	Rabbit	1:600	National Hormone and Peptide program, Dr. A.F.Parlow
Prolactin	AFP-879151	Rabbit	1:600	National Hormone and Peptide program, Dr. A.F.Parlow
Chromogranin A	ab15160	Rabbit	1:400	Abcam
Muc2 (Mucin)	SC-15334	Rabbit	1:200	Santa Cruz Biotechnology
Lysozyme	A0099 (EC 3.2.1.17)	Rabbit	1:1000	Dako
CDNF	DDV1	Rabbit	1:1000	(6)
CDNF	5187-CD	Rabbit	1:1000	R&D Systems

Importantly, anti-MANF antibodies have been validated by using *Manf*^−/−^ tissues. No background was detected in the tissues of *Manf*^−/−^ mice (Supplementary Figures 1H–J, 3A–M).

### Statistical Analysis

All data are presented as mean ± SEM. Statistical significance was assessed via Student's *t*-test between 2 groups as mentioned in the figure texts using GraphPad Prism 6 software (GraphPad Software, Inc., La Jolla, CA).

## Results

### MANF Expression During Embryonic Development in Mice

**Figure 1:**
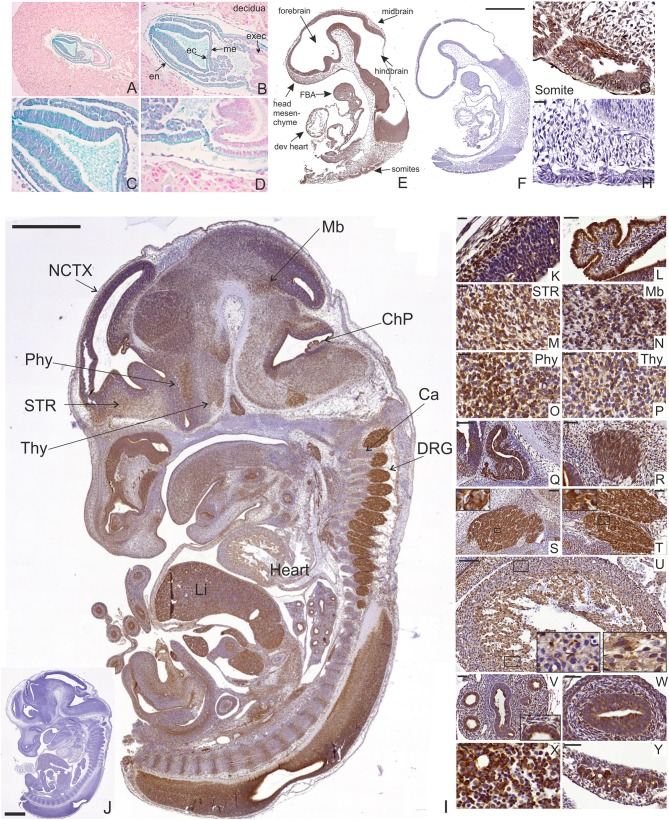
LacZ staining and MANF immunohistochemistry on sections from mouse embryos at E7.5, E9.5, and E13.5. **(A–D)** Positive LacZ staining observed in mesoderm (me), ectoderm (ec), and endoderm (en) layers of developing E7.5 heterozygous *Manf*^+/−^ mouse embryo. No positive LacZ staining was observed in extraembryonic ectoderm (exec). **(E–H)** MANF expression in developing E9.5 embryo **(E)**. MANF antibody did not stain E9.5 *Manf* ^−/−^ embryos, indicating high specificity of the used anti-MANF antibody **(F)**. MANF staining detected in the somites of E9.5 *Manf* ^+/+^ embryos **(G)**. No positive staining detected in *Manf* ^−/−^ somites **(H)**. Scale bar 500 μm **(E,F)**. Scale bar 20 μm **(G,H)**. dev. heart, developing heart; FBA, first branchial arch. **(I)** MANF expression in E13.5 mouse embryo with indicated anatomical structures. Scale bar 1,000 μm. No MANF positive signal was detected in E13.5 *Manf* ^−/−^ embryo **(J)**. Scale bar 1000 μm. **(K)** MANF antibody staining in the developing E13.5 cortex (CTX). **(L)** Strong MANF expression was detected in choroid plexus. MANF staining in developing striatum (STR) **(M)**, midbrain/pons junction (Mb) **(N)**, peduncular and terminal hypothalamic areas (Phy, Thy) **(O,P)**. Strong expression of MANF in the lumen of Rathke's pouch **(Q)**, vestibulocochlear ganglion **(R)**, trigeminal ganglia **(S)**, dorsal root ganglia (DRG) **(T)**. MANF expression in developing mouse heart **(U)**, developing mouse lung **(V)**, developing mouse gut **(W)**, developing mouse liver **(X)**. MANF expression in the developing pancreatic primordium **(Y)**. Scale Bar for 20 μm **(K,M-P,X)**. Scale Bar 50 μm for **(L,R,T,V,W,Y)**.

*Manf* mRNA expression has previously been analyzed by *in situ* hybridizations in E12.5 mouse embryos (39). Here we analyzed *Manf* gene expression in early embryonic development using staining for LacZ enzymatic activity of *Manf*^+/−^ embryos containing a β-galactosidase reporter gene (*lacZ*), which is controlled by the endogenous MANF promoter (16). We found LacZ staining indicating MANF expression in targeted *Manf*^+/−^ embryonic stem cells (ESC) (Supplementary Figures 1A,B). During early embryogenesis, LacZ staining was detected in E7.5 *Manf*^+/−^ embryos. LacZ negative decidual cells embedding the embryo are originating from the *Manf*^+/+^ mother (Figures 1A–D). Positive LacZ staining was detected in most cells of all three germ layers—ectoderm, endoderm and mesoderm in the E7.5 embryo (Figures 1A–D). Notably, no LacZ staining was detected in extraembryonic ectoderm, which contributes to the formation of the placenta (Figures 1A–D).

In order to analyze MANF expression pattern in mouse tissues, we used highly specific MANF antibodies, which we validated on *Manf* knockout tissue as a control for a negative signal. No background was detected in the tissues from *Manf*^−/−^ mice (Figures 1F,H,J, Supplementary Figures 1C–H, 3A–M).

Next, we studied the expression of MANF in E9.5 *Manf*^+/+^ and *Manf*^−/−^ embryos by IHC. Within the neural tube, MANF expression was observed in the regions forming the forebrain, midbrain, and hindbrain (Figure 1E). Outside the nervous system, MANF immunoreactivity cells were detected in somites giving rise to the dermis, skeletal muscles, and vertebrae (Figures 1E,G). MANF expression was also found in the developing heart, mandibular component of the first branchial arch and in the origin of second and third branchial arches in the E9.5 embryo (Figure 1E). The same positive signal was observed by LacZ in E9.5 heterozygous *Manf*^+/−^ mouse embryo (Supplementary Figures 1C,D). No staining was detected in *Manf*^−/−^ embryos (Figure 1F).

MANF was widely expressed in *Manf*^+/+^ embryos at E13.5 when many of the CNS and peripheral nervous system (PNS) structures, as well as other peripheral tissues, begin to differentiate (Figure 1I, Supplementary Figures 1E–G). In agreement with our previous *in situ* hybridization results, within the CNS, MANF immunostaining was observed in the neocortex, striatum, midbrain, hypothalamus and spinal cord (Figures 1I,K–P). High levels of MANF immunoreactivity was detected in the choroid plexus, the lumen of Rathke's pouch (Figure 1Q), an ectodermal structure forming the anterior pituitary gland, and vestibulocochlear ganglion (Figure 1R). In the PNS, MANF was highly expressed by neurons in trigeminal (Figure 1S) and dorsal root ganglia (Figure 1T). MANF immunoreactive cells were also observed in the developing peripheral tissues such as heart, lung, liver, intestine and developing pancreas (Figures 1U–Y) (5).

### Differential Expression Levels of MANF and CDNF in Adult Mouse Tissues

Our previous studies have demonstrated the wide distribution of *Manf* mRNA expression in the adult mouse brain by *in situ* hybridization, as well as protein expression in the adult cortex, hippocampus, cerebellum, striatum, and substantia nigra and non-neuronal tissues (39).

**Figure 2:**
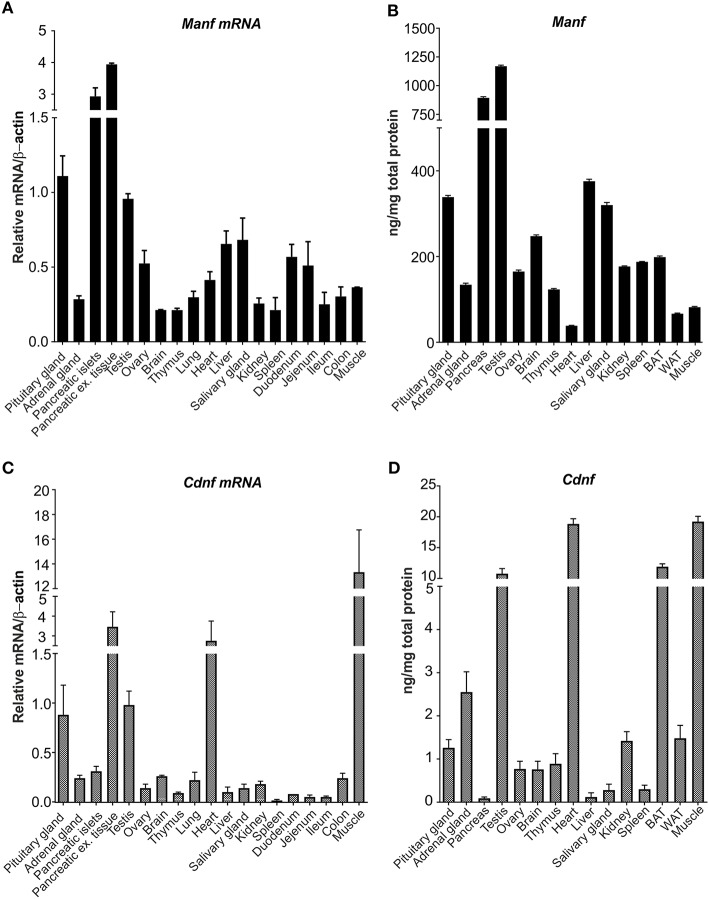
MANF and CDNF mRNA and protein expression in mouse brain and non-neuronal tissues. **(A)** RT-qPCR analysis of *Manf* mRNA expression in adult mouse brain and non-neuronal tissues. *n*-numbers for pituitary gland = 7, adrenal gland = 5, pancreases for islets = 8, pancreases for exocrine tissue, ovary, brain, lung, spleen, duodenum, jejenum, ileum, colon, muscle = 3, testis, heart and liver) = 4, thymus = 6. **(B)** Quantification of MANF protein in mouse tissues using mouse ELISA analysis, ng/mg total protein. *n*- numbers for pituitary gland, adrenal gland, testis, ovary, brain, thymus, heart, liver, salivary gland, kidney, brown adipose tissue and muscle) = 4, and for pancreas, spleen, and white adipose tissues) = 3. **(C)** RT-qPCR analysis of *Cdnf* mRNA expression in adult mouse brain and non-neuronal tissues. n-numbers for pituitary gland = 7, adrenal gland = 5, pancreases for islets = 8, pancreatic exocrine tissue, ovary, brain, lung, spleen, duodenum, jejenum, ileum, colon and muscle = 3, testis, heart and liver = 4, and thymus = 6. **(D)** CDNF mouse ELISA protein analysis in mouse tissues, ng/mg total protein. n-numbers for pituitary gland, adrenal gland, testis, ovary, brain, thymus, heart, liver, salivary gland, kidney, brown adipose tissue, and muscle = 4, pancreas, spleen, and white adipose tissue) = 3.

In this study, we analyzed and compared the levels of MANF and CDNF mRNA and protein expression in a broad panel of different tissues from adult mice using RT-qPCR and ELISA. Highest levels of *Manf* mRNA were observed in pituitary glands, endocrine islets of Langerhans, in the pancreas exocrine tissue separated from islets using collagenase digestion, and testis (Figure 2A) (5). Moderate levels of *Manf* mRNA expression was detected in ovary, heart, liver, and parts of intestine including duodenum and jejunum compared with low levels in the adrenal gland, brain, thymus, lung, salivary gland, kidney, spleen, ileum, colon, and muscle (Figure 2A).

Next, we analyzed the MANF protein expression by mouse MANF ELISA developed in our laboratory (42). In accordance with *Manf* mRNA levels, the highest concentrations of MANF protein was observed in pancreas and testis (Figure 2B). The MANF concentration in the pancreas was 894 ± 154 ng/mg total protein (*n* = 3), and in the testis 1,170 ± 168 ng/mg total protein (*n* = 4), respectively, indicating that MANF corresponds to ~1 promille of all proteins in these tissues. MANF concentration in the pituitary gland, liver, and salivary gland was between 300 and 400 ng/mg total protein, which is ~3 times lower compared to the MANF levels in testis and pancreas.

In the brain, brown adipose tissue, spleen, kidney, ovary, and thymus MANF protein concentration varied between 100 and 250 ng/mg total protein. The lowest MANF protein concentrations were found in skeletal muscle (82 ± 6 ng/mg total protein, *n* = 4), white adipose tissue (67 ± 4 ng/mg total protein, *n* = 3) and heart (38 ± 2 ng/mg total protein, *n* = 4). Protein concentration was almost 3-fold in brown adipose tissue (199 ± 14 ng/mg total protein, *n* = 4) compared to white adipose tissue.

Highest levels of *Cdnf* mRNA were found in the muscle, heart and exocrine tissue of pancreas by RT-qPCR (Figure 2C). Moderate levels of *Cdnf* mRNA expression were detected in pituitary gland and testis (Figure 2C). Low levels of *Cdnf* mRNA was observed in the other tissues studied (Figure 2C). Thus, the expression levels and tissue distribution of *Manf* mRNA differs from *Cdnf* mRNA in mouse tissues.

Consistent with *Cdnf* mRNA levels, highest levels of CDNF protein were observed in the skeletal muscle (19.2 ± 1.5 ng/mg of total protein, *n* = 4), heart (18.8 ± 1.4 ng/mg total protein, *n* = 4) and testis (10.8 ± 1.4 ng/mg total protein, *n* = 4) (Figure 2D). Interestingly, we observed high levels of CDNF protein also in brown adipose tissue (11.9 ± 0.4, ng/mg of total protein, *n* = 4). However, the median difference in CDNF and MANF concentration was ~125-fold in favor of MANF. The highest difference between MANF and CDNF concentrations was found in the pancreas (MANF vs. CDNF concentration 10 341x), liver (3 015x) and salivary gland (1 153x). In these tissues CDNF concentration was lowest measured (i.e., pancreas 0.09 ± 0.02 ng/mg total protein, *n* = 2; liver 0.12 ± 0.02 ng/mg total protein, *n* = 4; and salivary gland 0.28 ± 0.04 ng/mg total protein, *n* = 4, respectively). The smallest difference between MANF and CDNF concentrations was found in the heart (MANF vs. CDNF concentration 2.0 x), skeletal muscle (4.3x) and thyroid gland (4.5x). In these tissues, CDNF protein concentration was highest measured, while MANF concentration was at the lower end.

### MANF Expression in Structures of the Central and Peripheral Nervous Systems Responsible for Metabolism, Energy Homeostasis, and Hormonal Regulation

**Figure 3:**
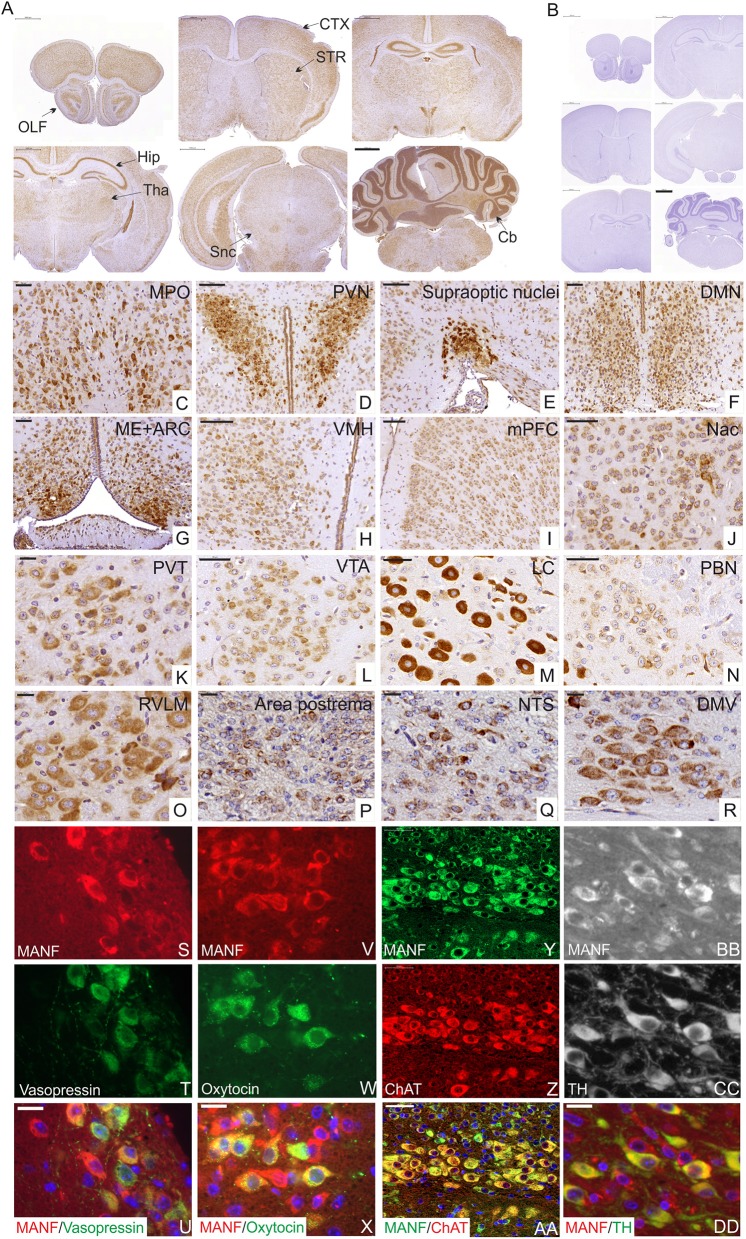
Immunohistochemical analysis of MANF expression in the mouse brain at postnatal day P14. **(A)** Low magnification of the mouse brain coronal sections stained with MANF antibody. **(A)** Scale bar 1,000 μm. No positive staining detected in *Manf* ^−/−^ mouse brain **(B)**. OLF, Olfactory bulb; CTX, cortex; STR, striatum; Hip, hippocampus; Tha, thalamus; Snc, substantia nigra pars -compacta; Cb, cerebellum. MANF expression was detected in most brain areas, with strong expression in homeostatic and hedonic control centers such as hypothalamus, dopamine mesolimbic system, and brainstem structures and neuroendocrine hypothalamic centers coordinating the hypothalamic pituitary endocrine axes. **(C–H)** Strong expression of MANF was detected in different parts of anterior and tubelar hypothalamus. **(C)** Medial preoptic nucleus (MPO). Scale bar 50 μm. **(D)** Paraventricular nuclei (PVN). Scale bar 100 μm. **(E)** Supraoptic nuclei. Scale bar 100 μm. **(F)** Dorsomedial hypothalamic nucleus (DMH). Scale bar 50 μm. **(G)** Medial eminence (ME) and arcuate nuclei (ARC). Scale bar 50 μm. **(H)** Ventromedial nuclei (VMH). Scale bar 50 μm. **(I–L)** MANF expression in dopamine mesolimbic system. **(I)** MANF expression in the medial prefrontal cortex (mPFC), Scale bar 100 μm. **(J)** Nuclei accumbens (Nac). Scale bar 50 μm. **(K)** MANF expression in paraventricular nucleus of the thalamus (PVT). Scale bar 50 μm. **(L)** Ventral tegmental area (VTA). Scale bar 20 μm. **(M–R)** MANF exression in the brainstem structures of the mouse brain. **(M)** Locus coeruleus (LC). Scale bar 50 μm. **(N)** Parabrachial nucleus (PBN). Scale bar 50 μm. **(O)** Rostral ventrolateral medulla (RVLM). Scale bar 20 μm. **(P)** Area postrema. Scale bar 20 μm. **(Q)** Nucleus of the solitary tract (NTS). Scale bar 20 μm. **(R)** Dorsal motor nucleus of Vagus (DMV). Scale bar 20 μm. **(S–DD)** Double immunohistochemistry analysis with anti-MANF antibody and antibodies against other peptide hormone and neuronal markers. MANF positive cells **(S,V,Y,BB)** were co-expressed with antidiuretic hormone vasopressin (green) **(T)** in supraoptic hypothalamic nucleus **(U)** and oxytocin (green) **(W)** in paraventricular hypothalamic nuclei **(X)**, with choline acetyltransferase positive neurons (ChAT) (red) **(Z)** in the dorsal motor nucleus of vagus **(AA)**, with tyrosine hydroxylase (green) (TH) **(CC)** positive neurons in ventral tegmental area **(DD)**. Cell nuclei were labeled with DAPI (blue). Scale bar, 20 μm.

The MANF protein expression pattern in the brain was studied using MANF antibody staining. Our results are consistent with our previous *in situ Manf* mRNA hybridization studies on P10 and adult mouse brain (39, 40). In this study, we analyzed in detail the expression of MANF in the juvenile brain of 2 week-old mice using MANF IHC. We observed MANF expression in all brain regions in the P14 mouse brain (Figures 3A–D, Supplementary Figures 2A–BB, Supplementary Text).

The hypothalamus is a key area of the brain known for the regulation of food intake, energy balance, and the coordination of the peripheral endocrine system through the pituitary gland (43, 44). Consistent with previous studies (5, 33, 39, 40), abundant expression of MANF was detected in different hypothalamic regions responsible for the hormonal regulation via adenohypophysis of the pituitary gland such as medial preoptic area (MPO) (Figure 3C) (release of gonadotrophin-releasing hormones), paraventricular hypothalamic nuclei (PVN) (Figure 3D) (secretion of somatostatin, growth-hormone-inhibiting hormone, corticotropin-releasing hormone, thyrotropin-releasing hormone), arcuate hypothalamic nucleus (ARC) (Figure 3G) (responsible for the secretion of growth hormone-releasing hormone and dopamine-mediated prolactin inhibition) and median eminence (functional link between the hypothalamus and pituitary gland) (Figure 3G). Magnocellular neurons of the PVN and supraoptic nuclei (SON) (Figure 3E) parts of hypothalamus are also responsible for the production of oxytocin and vasopressin that are released to the bloodstream via neurohypophysis of the pituitary gland. In this study we identified that MANF is co-expressed with oxytocin and vasopressin positive neurosecretory cells in the PVN and SON nuclei of the hypothalamus (Figures 3S–X), suggesting that proper amount of MANF expression may be needed for cells regulating processes such as sexual behavior, social recognition, stress response, depression, aging, and water balance of the body.

We also detected high MANF-expression in hypothalamic nuclei that are important for appetite, feeding and energy expenditure including ARC (contains appetite-stimulating (orexigenic neuropeptide Y and agouti-related peptide-expressing neurons) and appetite-suppressing neurons [anorexigenic pro-opiomelanocortin (POMC)-expressing neurons] (45), PVN, DMH (Figure 3F), LH and VMH (Figure 3H).

Another key brain structure involved in the regulation of food intake is the brainstem. We identified high levels of MANF expression in the locus coeruleus (LC) nucleus (Figure 3M), dorsal nucleus of the vagus nerve (DMNX) (Figure 3R), rostral ventrolateral medulla (RVLM) (Figure 3O) and low levels in parabrachial nucleus (PBN) (Figure 3N), area postrema (AP) (Figure 3P), sensory nucleus of the solitary tract (NTS) (Figure 3Q). Additionally, MANF expression was detected in circumventricular organs, such as the subcommissural organ (SCO) (Supplementary Figure 2L). Importantly, we detected MANF expression in choline acetyltransferase positive motor neurons of the dorsal nucleus of the vagus nerve (Figures 3Y–AA), which are known for their role in the regulation of gastrointestinal tract secretion, motility and in mediating pancreatic secretion (46).

In addition, the brain reward system is implicated in the control of hedonic feeding regulated by the mesolimbic and mesocortical dopaminergic pathways. Moderate expression levels of MANF was observed in the medial prefrontal cortex (mPFC) (Figure 3I), nucleus accumbens (NAc) (Figure 3J), paraventricular nucleus of the thalamus (PVT) (Figure 3K), and ventral tegmental area (VTA) (Figure 3L). We identified that MANF positive cells are co-expressed with tyrosine hydroxylase (TH)-positive dopamine neurons in the VTA (Figures 3BB–DD).

**Figure 4:**
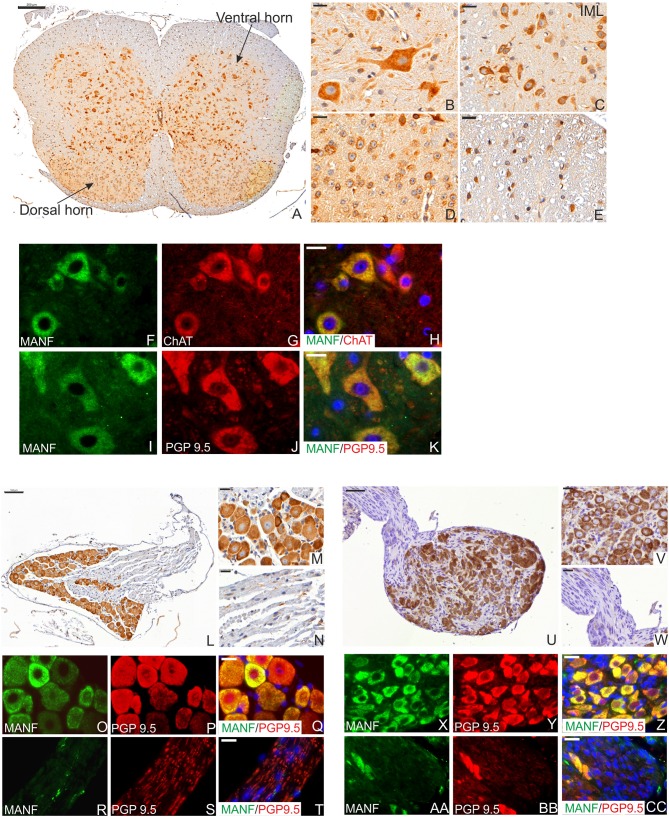
MANF expression in the mouse spinal cord and celiac ganglia at the postnatal day P56. **(A)** Low magnification of the mouse spinal cord coronal sections stained with MANF antibody. Scale bar, 200 μm. **(B)** Strong MANF expression was detected in neurons in the ventral horn of gray matter. **(C)** MANF expression in the neurons of intermediolateral column (IML) nucleus in dorsal horn of gray matter. **(D)** MANF expression in neurons in the central canal of gray matter. **(E)** Low expression of MANF was detected in the cells of white matter. **(B,C)** Scale bar, 20 μm. **(F–H)** Double immunohistochemistry analysis with anti-MANF **(F)** and choline acetyltransferase positive neurons (ChAT) antibodies **(G)**. MANF (green) was co-expressed with Vacht in motor neurons (red) in the ventral horn of gray matter **(H)**. MANF (green) was co-localized with PGP9.5 (red)-stained neurons in the ventral horn of gray matter **(K)**. **(F–K)** Scale bar, 20 μm. Cell nuclei were labeled with DAPI (blue). **(L)** Low magnification of the mouse dorsal root ganglia stained with MANF antibody. Scale bar, 100 μm. **(M)** Strong MANF immuno-reactivity was observed in the neuronal bodies. Scale bar, 20 μm but only few cells MANF-positive cells were detected in the nerve bundles of dorsal root ganglia **(N)**. Scale bar, 20 μm. **(O–T)** Double immunohistochemistry analysis with anti-MANF **(O,R)** and PGP9.5 antibodies **(P,S)**. MANF (green) was co-expressed with PGP9.5 (red) in neuronal bodies **(Q)**, but only scarce MANF expression was detected in the nerves of the celiac ganglia **(T)**. **(O–T)** Scale bar, 20 μm. **(U)** Low magnification of the mouse celiac ganglia stained with MANF antibody. Scale bar, 50 μm. **(V)** Strong MANF expression was observed in the neuronal bodies. Scale bar, 20 μm. **(W)** Negative MANF expression in the nerves of celiac ganglia. Scale bar, 20 μm. **(X–Z)** Double immunohistochemistry analysis with anti-MANF antibody **(X,AA)** and PGP9.5 positive neurons **(Y,BB)**. MANF (green) was co-expressed with PGP9.5 (red) positive neuronal bodies **(Z)**, but MANF expression was not detected in the nerves of the celiac ganglia **(CC)**. **(X–CC)** Scale bar, 20 μm.

Neurons of hypothalamus make projections onto neurons within the medulla that in turn project via the spinal cord through intermediolateral column (IML) to connect with the autonomic nervous system (ANS), thereby regulating heart rate, respiration, brown adipose tissues (BAT)/thermogenesis, and white adipose tissue (WAT) to modulate energy state. MANF expression was detected in neuronal bodies in the dorsal and ventral horns of the adult mouse spinal cord at P56 (Figures 4A–E). We also detected high MANF immunoreactivity in the IML nucleus (Figure 4C) of the spinal cord. MANF positive staining was observed in glial cells of the gray matter (Figure 4D) and in the myelinated axons of the white matter (Figure 4E). Very strong expression of MANF was detected in motor neurons and their dendrites immuno-labeled with choline acetyltransferase (Figures 4F–H) and neuronal and neuroendocrine protein gene product 9.5 (PGP9.5) markers (Figures 4I–K).

In the adult PNS, strong expression of MANF was observed within the sensory neuronal bodies of adult dorsal root ganglia (DRG) (Figures 4L–N) and in sympathetic celiac ganglia (CLG) (Figures 4U–W). BAT thermogenesis is regulated by a negative feedback loop via the DRG sympathetic nervous system stimulation (47). CLG is a part of the ANS which innervates most of the digestive tract, liver, and pancreas, thereby regulating their functions. Similarly to the spinal cord, only neuronal bodies were positive for MANF and not nerve fibers in DRG and CLG (Figures 4O–T,X–Z,AA–CC).

### Characterization of MANF Expression in the Mouse Endocrine System

**Figure 5:**
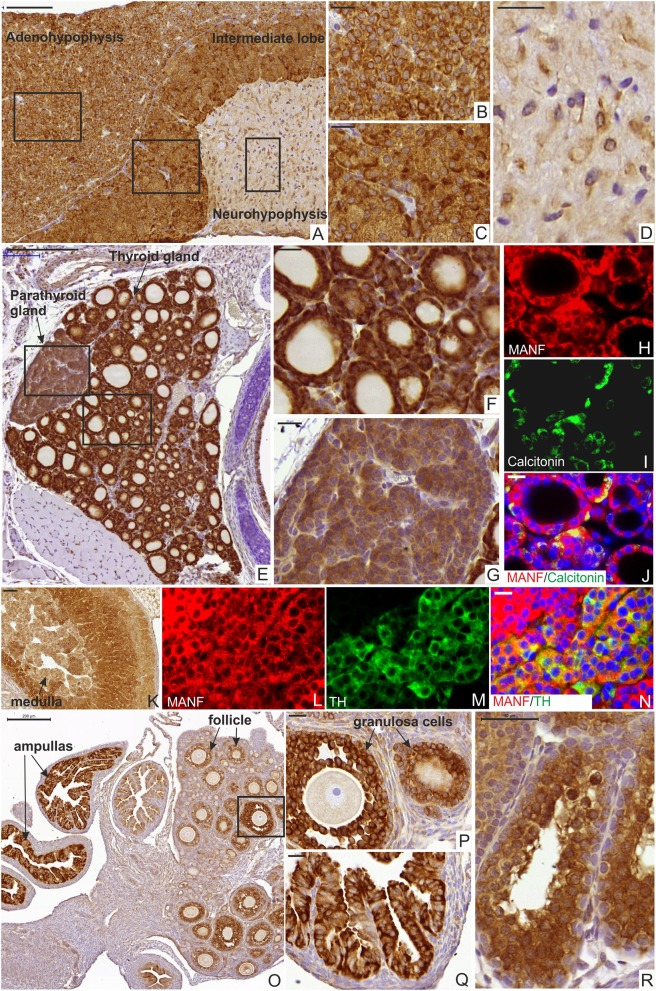
MANF expression in the mouse endocrine system. **(A)** Sagittal section of mouse pituitary gland at postnatal stage P56 stained with MANF antibody. Scale bar, 100 μm. **(B)** Strong MANF expression was observed in the adenohypophysis. Scale bar, 20 μm. **(C)** Strong MANF expression was observed in intermediate lobe of mouse pituitary gland. Scale bar, 20 μm. **(D)** Low expression of MANF was detected in the neurohypophysis. Scale bar, 20 μm. **(E)** Transversal section of the mouse thyroid gland at postnatal stage P14 stained with MANF antibody. Scale bar, 200 μm. **(F)** Strong MANF expression was observed in the follicular epithelium of the thyroid gland. Scale bar, 20 μm. **(G)** Strong MANF expression was detected in parathyroid gland. Scale bar, 50 μm. **(H–J)** Double immunohistochemistry analysis on thyroid section with anti-MANF **(H)** and anti-calcitonin **(I)** antibodies. Scale bar, 20 μm. MANF (red) was co-expressed with calcitonin (green) in C-cells **(J)**. **(K)** Strong MANF expression was detected in the cortex and moderate levels of MANF was observed in the medulla of mouse adrenal gland at postnatal stage P14. Scale bar, 50 μm. **(L–N)** Double immunohistochemistry analysis with anti-MANF antibody **(L)** and anti-TH **(M)** antibodies. MANF (red) was co-expressed with TH (green) in the medullar cells of the mouse adrenal gland **(N)**. Scale bar, 20 μm. **(O)** Low magnification of the mouse ovary at postnatal stage P14 stained with MANF antibody. Scale bar, 200 μm. **(P)** Strong MANF expression was observed in the granulosa cells surrounding the oocyte. Scale bar, 20 μm. **(Q)** Strong MANF expression was observed in the ampulla. Scale bar, 50 μm. **(R)** Strong MANF expression was detected in seminiferous tubules of the mouse testis at postnatal stage P14. Scale bar, 50 μm.

The endocrine system consists of the hypothalamus, pituitary gland, pineal body, thyroid and parathyroid glands, adrenal glands, and the reproductive organs (ovaries and testes). These glands secrete hormones to the blood circulation, which control cellular and organ growth, development, metabolism, reproduction, sleep, and response to stress stimuli among others. Incorrect amount of hormone production leads to medical conditions such as insulin-deficiency in Type 1 diabetes, growth hormone-deficiency in postnatal growth defects caused by hypopituitarism and hormonal dysregulation in metabolic syndrome (43, 48, 49). Our qPCR and ELISA results for MANF expression showed that MANF is highly expressed in mouse endocrine tissues such as the pituitary gland, the thyroid gland, pancreatic islets and testis as well as exocrine tissues such as exocrine pancreas, salivary glands and other tissues containing cells with secretory function (Supplementary Text and Supplementary Figure 4) compared to the rest of the tissues (Supplementary Text and Supplementary Figures 5, 6). Therefore, we studied the cellular distribution of MANF expression by IHC in different juvenile and adult mouse endocrine organs (Figure 5). In the adult mouse pituitary gland, strong MANF immunoreactivity was observed in most cells of the endocrine anterior adenohypophysis (Figures 5A,B) and the intermediate lobe (Figures 5A,C) as well as in cells of the neurohypophysis (Figures 5A,D). Additionally, we found strong MANF expression in the thyroid follicular cells producing thyroxine (T4) and triiodothyronine (T3) (Figures 5E,F). MANF was also co-expressed with calcitonin in parafollicular C-cells, responsible for reducing calcium levels in the blood by inhibiting osteoclast activity in bones and inhibiting renal reabsorption (Figures 5H–J). MANF immunoreactivity was strong also in parathyroid glands (Figures 5E,G), that is responsible for the production of parathyroid hormone and thereby regulation of calcium and phosphate levels in the blood. In the adrenal gland, in contrast to moderate and low levels of MANF mRNA and protein detected by RT-qPCR and ELISA (Figures 2A,C), intense MANF staining was detected in the cortex and less in the medulla (Figure 5K). Furthermore, MANF was co-expressed with TH in the medullar chromaffin cells, responsible for the synthesis and release of catecholamine, adrenaline, and noradrenaline, in response to stress (Figures 5L–N).

In the mouse reproductive system, we observed MANF expression in the mouse ovaries and ampullas of the Fallopian tubes (Figures 5O–Q). MANF expression was detected in granulosa or cumulus cells, which surround and nourish the oocytes in the ovary (Figure 5P). Additionally, we detected MANF expression in male seminiferous tubules of the mouse testis at P14 (Figure 5R).

### Ablation of MANF Expression in *Manf ^−/−^* Mice Leads to Defects in the Anterior Pituitary Gland

The phenotype of *Manf*^−/−^ mice is characterized by the progressive loss of circulating insulin leading to diabetes and a severe growth defect (16). We have previously demonstrated that the dramatic growth retardation in conventional *Manf*^−/−^ mice is not dependent on the diabetic phenotype as the growth defect is not recapitulated in the pancreas-specific *Pdx-1Cre::Manf*^*fl*/*fl*^ mice, that develop diabetes but no growth retardation (16). In addition, neuron-specific *NestinCre*^+/−^*::Manf*^*fl*/*fl*^ mice do not develop an equally severe growth defect compared to global *Manf*^−/−^ mice [(16); Pakarinen et al. submitted manuscript]. Short stature or dwarfism is usually caused by a pituitary growth-hormone deficiency in humans (50). Thus, the short stature of *Manf*^−/−^ mice and the high MANF expression in the pituitary gland prompted us to study the pituitary gland in more detail in the *Manf*^−/−^ mice.

**Figure 6:**
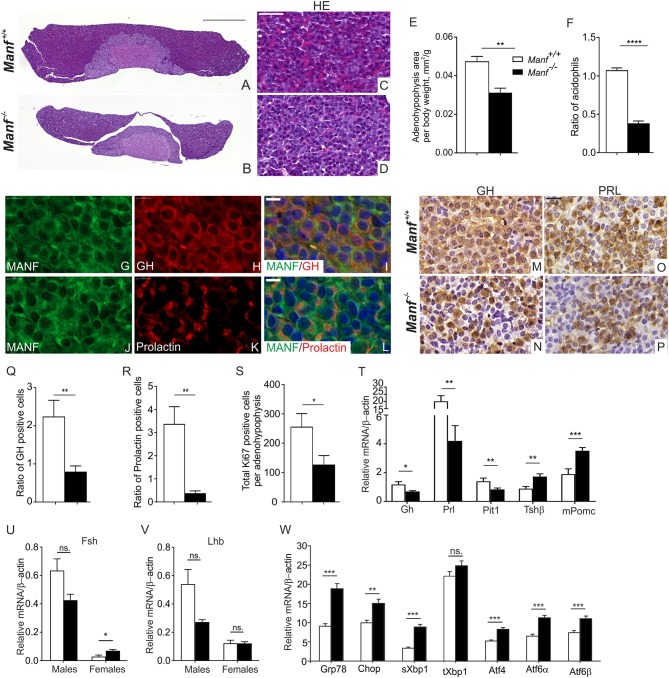
Ablation of MANF in the conventional *Manf* ^−/−^ mice results in abnormal development of the adenohypophysis in the pituitary gland. **(A–D)** Hematoxylin and eosin staining of mouse pituitary gland from *Manf*^+/+^ **(A,C)** and *Manf*^−/−^ **(B,D)** mice at postnatal stage P42. **(A,B)** Scale bar, 500 μm. **(C,D)** Scale bar, 50 μm. **(E)** Area of adenohypophysis normalized to body weight of the animal (mm^2^/g). Adenohypophysis area of *Manf*^−/−^ (black bars, *n* = 6) pituitary glands was significantly reduced compared to *Manf*^+/+^ (white bars, *n* = 5) mice at 6 weeks of age. **(F)** Ratio of granule-filled acidophilic cells (purple) representing somatotropes and lactotropes compared to basophils (violet) was significantly reduced in the anterior lobe of *Manf*^−/−^ pituitaries (*n* = 6) compared to those of *Manf*^+/+^ mice (*n* = 5). **(G–L)** Double immunohistochemistry analysis with anti-MANF antibody **(G,J)** and antibodies against growth hormone (GH) **(H)** and prolactin **(K)** expressing cells. MANF (red) was co-expressed with GH and prolactin (green) cells of the adenohypophysis **(I,L)**. Cell nuclei were labeled with DAPI (blue). Scale bar, 20 μm. **(M–P)** Growth hormone (GH) **(M,N)** and Prolactin (PRL) **(O,P)** immunohistochemistry on sections from adenohypophyses from *Manf*^+/+^ (*n* = 4) **(M,O)** and *Manf*^−/−^ (*n* = 5) **(N,P)** mice at postnatal stage P42. Scale bar, 20 μm. **(Q,R)** Ratio of GH- **(Q)** and PRL- **(R)** positive cells was significantly reduced in the adenohypophyses of *Manf*^+/+^ (*n* = 4) and *Manf*^−/−^ (*n* = 5) mice at postnatal stage P42. **(S)** Proliferation of cells in the adenohypophyses assessed by Ki67-positive cells compared to total amount of cells, *n*- numbers; *Manf*^+/+^ mice= 4, *Manf*^−/−^ mice= 5. **(T–V)** Quantitative RT-qPCR analysis for expression levels of hormonal genes in the mouse pituitary gland from *Manf*^+/+^ (white bars) and *Manf*^−/−^ mice (black bars) at postnatal age P42. *n* = 9 mice per group. For *Fsh* **(U)** nad *Lhd* **(V)** mRNA levels. *Manf*^+/+^ male mice (*n* = 3), *Manf*^−/−^ male mice (*n* = 5), *Manf*^+/+^ female mice (*n* = 6), *Manf*^−/−^ female mice (*n* = 5). **(W)** Quantitative RT-qPCR PCR analysis of UPR genes in the pituitary glands from *Manf*^+/+^ (*n* = 10) (white bars) and *Manf*^−/−^ (*n* = 11) (black bars) mice at postnatal age P42. Mean ± SEM, *p < 0.05, **p < 0.01, ***p < 0.001, ****p < 0.0001 vs. the corresponding control.

The pituitary gland plays crucial roles in growth, reproduction, endocrine functioning, and functions to convey signals from the hypothalamus to various organs (43, 51). The endocrine anterior pituitary gland contains five types of cells, each secreting different hormones, where somatotropes secrete growth hormone (GH), lactotropes prolactin (PRL), gonadotropes follicle stimulating hormone (FSH) and luteinizing hormone (LH), corticotropes adrenocorticotropic hormone (ACTH), and thyrotropes thyroid-stimulating hormone (TSH) (43). We stained sections from the pituitary glands with hematoxylin-eosin for morphological analysis (Figures 6A,B). This revealed that the size of the adenohypophysis in *Manf*^−/−^ mice was reduced compared to the adenohypophysis from *Manf*^+/+^ mice in relation to body weight at 6 weeks of age (Figure 6E), while the area of the intermediate lobe was not changed between the groups (Supplementary Figure 7A) and the area of neurohypophysis was increased in *Manf*^−/−^ mice compared to control (Supplementary Figure 7B). The ratio of granule-filled acidophilic cells (purple) representing somatotropes and lactotropes (Figures 6C,D) compared to basophils (blue) were significantly reduced in the anterior lobe of 6-week-old *Manf*^−/−^ mice compared to *Manf*^+/+^ mice (Figure 6F). Double immunofluorescence staining revealed co-localization of MANF with growth hormone (Figures 6G–I) and prolactin (Figures 6J–L) in cells of the anterior pituitary lobe at 6 weeks of age.

Immunoperoxidase staining of the pituitary glands revealed decreased GH (Figure 6N) and PRL (Figure 6P) expression in the adenohypophyses of the *Manf*^−/−^ mice compared to *Manf*^+/+^ mice (Figures 6M,O). Quantification of the ratio of GH- and PRL-positive cells in the adenohypophyses confirmed a severely reduced number of GH (Figure 6Q) and PRL (Figure 6R) producing cells in *Manf*^−/−^ mice compared to that of *Manf*^+/+^ mice. In line with the reduced size of the adenohypophysis in *Manf*^−/−^ mice, we found a clear reduction in the number of proliferative cells (Figure 6S, Supplementary Figures 7C,D) but no increase in the number of dying cells (Supplementary Figure 7E) in the adenohypophyses of *Manf*^−/−^ mice.

Consistent with the reduced number of GH and PRL positive cells, mRNA expression levels of *Gh* and *Prl* (Figure 6T) genes were significantly decreased in the pituitaries isolated from *Manf*^−/−^ mice compared to those of *Manf*^+/+^ mice regardless of gender. The levels of pro-opiomelanocortin *(Pomc)* mRNA and Thyroid-stimulating hormone (*Tsh*) mRNA were significantly increased in *Manf*^−/−^ pituitaries (Figure 6T), probably reflecting the higher relative number of corticotropes and thyrotropes in the *Manf*^−/−^ pituitaries. In accordance with the essential role of the pituitary-specific positive transcription factor 1 (*Pit1*) for the development of somatotropes and lactotropes, we observed that *Pit1* mRNA expression was significantly downregulated in the *Manf*^−/−^ mutant pituitary glands (Figure 6T). Both mRNA levels for luteinizing hormone (*Lh)* and follicle-stimulating hormone *(Fsh)* were non-significantly downregulated in the pituitaries of 6 weeks old *Manf*^−/−^ male mice compared to *Manf*^+/+^ controls (Figures 6U,V), while *Fsh* mRNA levels were significantly upregulated and *Lh* mRNA was not affected in pituitaries of *Manf*^−/−^ female mice.

As chronic ER stress precedes the reduced pancreatic β-cells proliferation and increased β-cells death leading to diabetes in *Manf*^−/−^ mice (16), we investigated whether ablation of MANF from the pituitary leads to ER stress and chronic UPR activation. Importantly, we found significant upregulation of UPR genes representing all three main UPR pathways PERK, IRE1α, and ATF6 in the pituitaries isolated from 6 week-old *Manf*^−/−^ mice indicating that chronic ER stress may be a cause of the acidophilic cell loss in the pituitary glands of *Manf*^−/−^ mice (Figure 6W).

Hence, these results suggest that MANF expression is needed to support the GH and PRL expressing cells in the anterior pituitary of mice.

## Discussion

In summary, this study provides a systematic analysis of MANF expression in mouse tissues with a focus on neuronal and peripheral tissues, including cells with high metabolic, and secretory function by different techniques. We provide a comparative analysis of MANF and CDNF mRNA and protein expression in postnatal mouse tissues (Tables 2, 3). The strength of our expression analysis lies in the validation of the specificity of the techniques using tissue-samples from *Manf*^−/−^ and *Cdnf*^−/−^ mice. In addition, we have confirmed the reporter β-galactosidase expression under the *Manf* promoter in tissues of heterozygote and homozygote *Manf*^+/−^ and *Manf*^−/−^ mice using X-gal staining. Importantly, we report a novel pituitary gland phenotype in the MANF-deficient mice, which might contribute to the severe postnatal growth defect. We are the first to report MANF expression in mouse embryonic stem cells, as well as in all three germ layers during mouse gastrulation. This it is not surprising since MANF is ubiquitously expressed in most developing organ systems in the embryo and through adulthood. In the postnatal CNS, MANF expression was very high in the cells of the various areas that regulate energy metabolism and the endocrine system including the mesocortical/mesolimbic dopamine system, different areas of hypothalamus, and brainstem structures of the mouse brain. In a recent study, increased expression of MANF in hypothalamic neurons of mice led to increased feeding behavior and obesity, whereas reduced expression specifically in hypothalamic neurons led to hypophagia and reduced body weight (33). The mechanism behind MANF action was suggested to rely on its ability to regulate insulin signaling by interacting with phosphatidylinositol 5-phosphate 4-kinase type-2 beta (PIP4K2B) in the ER and thus regulating AKT phosphorylation and insulin sensitivity (33). In addition, reduced MANF expression in tissues and sera of mice led to increased liver inflammation, fibrosis, and steatosis whereas MANF supplementation seemed to reverse age-dependent inflammatory and metabolic changes and induce rejuvenation in mice (34). In the fruit fly, overexpression of MANF in cells of peripheral tissues led to a significant extension of life-span, whereas overexpression of MANF in the brain led to reduced life-span (34). These results suggest that proper regulation of MANF expression in the brain and other organs is important to meet the metabolic demand. Thus, the exact mechanism of MANF action in metabolic regulation remains to be elucidated.

**Table 2:** Table of MANF expression in mouse tissues assessed by different techniques.

**Mouse tissue**	**qRT-PCR Relative *Manf* mRNA level/β-actin**	**ELISA MANF protein levels, ng/mg total protein**	**IHC MANF**
Pituitary gland	+ + + +	+ + + +	+ + + + +
Thyroid gland	+ +	+ +	+ + + + +
Adrenal gland	+ +	+ +	+ + + +
Pancreas	Not analyzed	+ + + + +	+ + + + +
Islets	+ + + + +	Not analyzed	+ + + + +
Exocrine tissue	+ + + + +	Not analyzed	+ + + + +
Testis	+ + + +	+ + + + +	+ + + + +
Ovary	+ + +	+ +	+ + +
Brain	+	+ + +	+ + +
Thymus	+	+	+ +
Lung	+ +	Not analyzed	+
Heart	+ +	+	+
Liver	+ + +	+ + + +	+ + +
Salivary gland	+ + +	+ + + +	+ + + + +
Kidney	+	+ +	+
Spleen	+	+ +	+
Duodenum	+ + +	Not analyzed	+ + +
Jejunum	+ + +	Not analyzed	Not analyzed
Ileum	+ +	Not analyzed	Not analyzed
Colon	+ +	Not analyzed	Not analyzed
Muscle	+ +	+	+
Brown adipose tissue (BAT)	Not analyzed	+ + +	+ +
White adipose tissue (WAT)	Not analyzed	+	+

**Table 3:** MANF expression in mouse tissues.

**Mouse tissue**	**qRT-PCR Relative *Manf* mRNA level/β-actin**	**ELISA MANF protein levels, ng/mg total protein**	**IHC MANF**
Pituitary gland	P56	P56	P56
Thyroid gland	P56	P56	P14
Adrenal gland	P56	P56	P14
Pancreas	Not analyzed	P56	P1, P14, P56 (5)
Islets	P56 (5)	Not analyzed	P1, P14, P56 (5)
Exocrine tissue	P56 (5)	Not analyzed	P1, P14, P56 (5)
Testis	P56	P56	P14
Ovary	P56	P56	P14
Brain	P56	P56	P14
Thymus	P56	P56	P14
Lung	P56	Not analyzed	P14
Heart	P56	P56	P14
Liver	P56	P56	P14
Salivary gland	P56	P56	P56
Kidney	P56	P56	P14
Spleen	P56	P56	P14
Duodenum	P56	Not analyzed	P14
Jejunum	P56	Not analyzed	Not analyzed
Ileum	P56	Not analyzed	Not analyzed
Colon	P56	Not analyzed	Not analyzed
Muscle	P56	P56	P14
Brown adipose tissue (BAT)	Not analyzed	P56	P14
White adipose tissue (WAT)	Not analyzed	P56	P14
Spinal Cord	Not analyzed	Not analyzed	P56
Dorsal root ganglia (DRG)	Not analyzed	Not analyzed	P56
Celiac ganglia (CLG)	Not analyzed	Not analyzed	P56

In previous studies by us and others it has been demonstrated that MANF is highly expressed in secretory tissues including mouse and human pancreatic exocrine acinar cells and β-cells of Langerhans islets and mouse salivary gland and testis (4, 5, 16, 39, 52). In this study, we show for the first time that MANF is highly expressed also in the tissues of mouse endocrine system such as in the pituitary, thyroid, parathyroid, and adrenal glands as well as in the mouse female reproductive system. We found strong expression of MANF in the thyroid follicular and para-follicular cells that produce vital hormones for all body functions. Histologically the thyroid gland in the *Manf*^−/−^ mice appears normal (Danilova, Lindahl, unpublished results). However, it remains to be determined whether the circulating levels of calcitonin, T4 and T3 and parathyroid hormone are reduced in MANF-deficient mice, which could, in addition to diabetes contribute to the retarded growth and reduced life-span in *Manf*^−/−^ mice (16).

In the mouse adrenal gland, MANF expression was high in cells of the zona glomerulosa of the cortex that is responsible for the production of mineralocorticoids such as aldosterone. In accordance, high levels of MANF was found in cells of the juxtaglomerular apparatus in the mouse kidney, indicating that MANF expression levels could be important for the proper function of the renin-angiotensin–aldosterone hormonal system required for control of the blood pressure and sodium homeostasis. High MANF expression was also found in cells producing glucocorticoids, which increase glucose production from amino acids in the liver and increase the anti-inflammatory response. In addition, MANF was expressed in the adrenal zona reticularis, which produces androgens that are converted to sex hormones in the gonads. In accordance, strong expression of MANF was observed in the female and male mouse reproductive organs. In the female uterine tube, high MANF expression was observed in the epithelium of the fallopian tubules. In addition, MANF was highly expressed by granulosa cells, especially in cells surrounding some oocytes, suggesting that MANF levels are increased with oocyte maturation. In addition, MANF was highly expressed in differentiating spermatocytes in the mouse seminiferous tubules in the testes. Consistent with our mouse studies, the *Manf-1* gene was activated in the spermatheca of transgenic *C. elegans* (53). Interestingly, MANF-deficient *C. elegans* displayed fewer offspring due to fewer fertilized eggs being laid than their wild type littermates (53, 54). Additionally, *in vitro* maturation procedure led to enhanced MANF expression levels in the mouse oocytes (55), possibly due to the stress caused by the procedure. The heterozygous *Manf*^+/−^ female and male mice are fertile and breed normally (16). However, diabetic female and male *Manf*^−/−^ mice do not produce offspring suggesting that they are infertile (Lindahl, personal observation). Therefore, MANF expression might be needed for the highly metabolic action of cells in both female and male mouse fertility processes. The mechanism of MANF action in the mouse reproductive system remains to be studied. To conclude, the pattern and level of MANF expression in the neuroendocrine and endocrine mouse systems suggest important roles for MANF in cells with a high level of hormonal secretory function within the hypothalamus-pituitary-thyroid/adrenal/gonadal axes.

We compared the MANF expression pattern and levels observed in mouse tissues with the expression data found from human tissue in the Human Protein Atlas database (https://www.proteinatlas.org/ENSG00000145050-MANF/tissue). Similarly to the mouse brain, MANF was high in neurons but low or non-existing in glial and endothelial cells in the human brain. In endocrine glandular cells of the human thyroid and adrenal glands and islets of Langerhans, MANF levels were moderate or high. Similarly to glandular cells in mouse tissues, MANF expression was high in glandular cells of the exocrine pancreas, cells in seminiferous ducts, and fallopian tubules. In human kidney, MANF expression was moderate in the tubules and low in the glomeruli. In human liver hepatocytes, MANF expression was high in accordance with data for mouse. In contrast to our mouse data, MANF levels were low or non-existing in human salivary gland, but similarly to mouse MANF expression was low in human heart and muscle. In conclusion, similar high MANF expression was detected in glandular cells of human tissues compared to our mouse data, suggesting the importance of MANF in both human and mouse for cells with high protein synthesis and secretion.

In order to provide the clues for potentially distinct activities of MANF and CDNF, we studied MANF and CDNF mRNA and protein expression levels in mouse tissues. We observed the expression of MANF and CDNF in all tissues that were analyzed. Notably, CDNF protein levels appear generally lower than MANF protein levels in mouse tissues. While high levels of MANF was detected within mouse endocrine and exocrine glands, similarly to previous studies (6), the highest levels of CDNF was observed in the tissues with high-energy demands and oxidative roles including heart, muscle, testis, and BAT.

A number of studies have indicated that exogenous MANF is protective for cells in different pathological conditions including neurodegenerative, metabolic, and inflammatory diseases (14, 34). We have previously demonstrated vital roles for endogenous MANF in pancreatic β-cells (5, 16). In this study, we explore new roles for MANF in the pituitary gland. We found that MANF was highly expressed already in the developing anterior pituitary gland, the ectodermal derived Rathke's pouch in mice at E13.5. In adult mice, MANF was highly expressed in anterior and intermediate parts of the pituitary gland. Previously we demonstrated that global MANF-deficiency in *Manf*^−/−^ mice resulted in a severe growth retardation, including a 40% reduction in weight at 8 weeks of age compared to *Manf*^+/+^ mice (16). As growth retardation in human is associated with hypopituitarism and growth hormone-deficiency (50), we set out to study the effect of MANF-removal from the pituitary gland in mice. Unexpectedly, we found that the size of the anterior pituitary gland was reduced in *Manf*^−/−^ mice probably due to the decreased number of GH and PRL-producing cells and reduced number of proliferating cells in the anterior pituitary. The reason for the reduced proliferation was most likely due to severe ER stress activating all three major UPR branches in the *Manf*^−/−^ pituitary glands. In addition, mRNA levels for *Gh* and *Prl* genes were reduced and proportionally the levels of *Tsh*β and *Pomc* transcripts were increased, confirming the reduced number of somatotropes and lactotropes in *Manf*^−/−^ anterior pituitary gland. The reduced number of GH-expressing cells as well as reduced proportional levels of *Gh* mRNA expression would suggest reduced levels of circulating GH in the *Manf*^−/^^−^ mice thus contributing to the growth defect. GH is important for tissue growth and remodeling through inducing the expression of insulin growth factor-1 mRNA in the liver leading to increased release of IGF-1 into the bloodstream (56). However, the growth retardation in GH-deficient mutant mice starts only 2 weeks after birth (57), whereas *Igf1*^−/−^ mice are 35% smaller than wild-type mice at birth (58). As *Manf*^−/−^ mice are already significantly smaller than their wild-type litter-mates at E18.5 before birth (16), the growth retardation in *Manf*^−/−^ mice cannot solely be due to reduced GH expression.

A recent study has identified the importance of MANF for skeletal growth (59). Cartilage-specific ablation of *Manf* in mice led to skeletal dysplasias caused by reduced chondrocyte proliferation and increased ER stress. However, the skeletal MANF-deficiency in these mice resulted in only a very mild defect in long bone growth (about 5%), while the body length in *Manf*^−/−^ mice was reduced by 16% at 8 weeks of age. Interestingly, *Perk*^−/−^ mice were found normal at birth but showed a severe postnatal growth retardation, skeletal dysplasias and osteopenia followed by a loss of endocrine and exocrine functions of the pancreas (25). Interestingly, the growth retardation was found to be caused by decreased neonatal *Igf-1* expression in the liver but not from other tissues, leading to reduced circulating IGF-1 serum levels important for neonatal growth (60). It has been speculated that PERK may be required in the liver for the translational initiation of one or more factors that control the neonatal expression of *Igf-1* mRNA. As both IGF-1 and IGF-2 are important for embryonic growth (61), MANF-deficiency might affect the expression of these growth factors or proteins in general through increased ER stress leading to PERK activation, increased eIF2α phosphorylation and attenuation of translational initiation thus attenuating cell proliferation and growth.

Our findings show that MANF is widely expressed in different cells and tissues in the mouse, but its expression is especially high in glandular and metabolically active cells with high synthesis of secreted proteins. The expression levels of MANF seems to be cell-specific, tightly regulated, and conserved in human and mouse. Lack of MANF expression in MANF conventional knockout mice leads to ER stress in pituitary gland resulting in reduced proliferation and loss of GH- and PRL expressing endocrine cells thereby probably contributing to the retarded growth in *Manf*^−/−^ mice. Thus, cells under higher basal ER stress might require more MANF for proper protein folding to maintain the ER homeostasis.

## Data Availability Statement

The datasets generated for this study are available on request to the corresponding author.

## Ethics Statement

This study was carried out in accordance with Finnish Animal Ethics Committee of the State Provincial Office of Southern Finland. Approval numbers - ESAVI/11865/04.10.07/2017, ESAVI/3117./04.10.07/2017.

## Author Contributions

TD designed and performed experiments, constructed figures, and wrote the manuscript. EG designed, performed MANF and CDNF mouse ELISA's, and contributed to the writing of the manuscript. EPak contributed to the interpretation of the results. EPal isolated the mouse spinal cords and performed MANF/ChAT double stainings. PL designed MANF and CDNF mouse ELISA's. MS designed experiments and provided funding. ML created the MANF- and CDNF-deficient mice, designed and performed experiments, provided funding, and wrote the manuscript. TD, EG, PL, MS, and ML revised the manuscript. All authors commented on the manuscript and accepted the final version of the manuscript.

### Conflict of Interest

The authors declare that the research was conducted in the absence of any commercial or financial relationships that could be construed as a potential conflict of interest.
