# Supplementary material for: Mesencephalic Astrocyte-Derived Neurotrophic Factor (MANF) Is Highly Expressed in Mouse Tissues With Metabolic Function
Source: Front Endocrinol (Lausanne). 2019 Nov 6;10:765. doi: 10.3389/fendo.2019.00765 (PMC6851024; doi:10.3389/fendo.2019.00765)
Supplement: Supplementary file 1 [file Presentation_1.pdf]

Supplementary materials

Supplementary text

MANF expression in CNS

We detected wide MANF expression in the olfactory bulb, cortex, striatum, hippocampus, thalamus, choroid plexus, substantia nigra and cerebellum (Figure 3A, Supplementary Figure 2A-K). In accordance with previous studies (Lindholm et al., 2008; Yang et al., 2014; Tseng et al., 2017), we observed MANF positive immuno-reactivity in the NeuN positive neuronal cells in the cortex (Supplementary Figure 2Q-S), but not in GFAP positive astrocytes in hippocampus (Supplementary Figure 2D-F). MANF was co-expressed with TH-positive dopamine neurons in the substantia nigra (Supplementary Figure 2W-Y), and calbindin-positive Purkinje cells in the cerebellum (Figure 2Z-BB).

MANF expression in other organs

Besides CNS, PNS and the major endocrine mouse systems, we also observed high MANF expression in organs containing cells with high secretory function. In the exocrine salivary glands, MANF was highly expressed in the sublingual gland (Supplementary Figure 4A), submandibular gland (Supplementary Figure 4B) and parotid gland (Supplementary Figure 4C), all containing cells that secrete saliva containing serous alpha-amylase and mucous mucin (Supplementary Figure 4A-C).

In the normal mouse kidney, high levels of MANF immuno-reactivity was detected within the glomerulus, possibly in the cells of juxtaglomerular apparatus involved in renin production and regulation of blood pressure. Low levels of MANF was detected in renal tubular cells in the kidney (Supplementary Figure 4D-G). In the mouse liver at P14, moderate MANF expression was detected in hepatocytes (Supplementary Figure 4G).

The lung contains a mixture of epithelial, endothelial and hematopoietic cells. We observed variable expression of MANF in the cells of mouse lungs at P14 (Supplementary Figure 4H). Very high MANF staining was observed in tracheal (Supplementary Figure 4I) and in the esophagus (Supplementary Figure 4J) epithelial cells. In addition, we observed MANF expression in the skin

epithelium, sebaceous glands and the cells of the dermis of the mouse skin (Supplementary Figure 4K).

We also observed MANF expression throughout the gastrointestinal (GI) system. Particularly, MANF immunoreactivity was detected in epithelial cells of the villi (Supplementary Figure 4L), enterocytes stained with anti-chromogranin A antibody (Supplementary Figure 4M-O) and goblet cells expressing mucin (Supplementary Figure 4P-R) in the duodenum. MANF was also found expressed in crypt Paneth cells shown by anti-lysozyme staining (Supplementary Figure 4S-V).

Within the cardiovascular and muscular systems, a limited amount of MANF positive cells were found in cardiac muscle and atrium tissue of the mouse heart (Supplementary Figure 5A-C) and in the muscle (Supplementary Figure 5E). MANF immunoreactive cells were also detected in epicardial adipose tissue (Supplementary Figure 5D). Interestingly, we also detected MANF positive cells in adipocytes of WAT (Supplementary Figure 5F) and BAT (Supplementary Figure 5G).

Within the mouse lymphatic system including spleen, thymus, lymph node, and Peyer's patch, MANF expression was moderate in spleen and thymus according to *Manf* mRNA levels (Figure 2A) and MANF protein levels analyzed by ELISA and IHC (Figure 2A and 2B, Supplementary Figure 6A-H). Consistent with previous studies (Liu et al., 2015), MANF expression was predominantly found within the red pulp and marginal-zone in the mouse spleen (Supplementary Figure 6A-B). In the mouse thymus, MANF expression was mainly detected in the medulla (Supplementary Figure 6C-D). Positive MANF cells were also observed in mouse lymph node (Supplementary Figure 6E-F) and small lymphatic tissue of the intestine Peyer's patch (Supplementary Figure 6G-H).

References

Lindholm, P., Peranen, J., Andressoo, J.O., Kalkkinen, N., Kokaia, Z., Lindvall, O., et al. (2008). MANF is widely expressed in mammalian tissues and differently regulated after ischemic and epileptic insults in rodent brain. *Mol Cell Neurosci* 39(3), 356-371. doi: 10.1016/j.mcn.2008.07.016.

Liu, J., Zhou, C., Tao, X., Feng, L., Wang, X., Chen, L., et al. (2015). ER stress-inducible protein MANF selectively expresses in human spleen. *Hum Immunol* 76(11), 823-830. doi: 10.1016/j.humimm.2015.09.043.

Tseng, K.Y., Danilova, T., Domanskyi, A., Saarma, M., Lindahl, M., and Airavaara, M. (2017). MANF Is Essential for Neurite Extension and Neuronal Migration in the Developing Cortex. *eNeuro* 4(5). doi: 10.1523/ENEURO.0214-17.2017.

Yang, S., Huang, S., Gaertig, M.A., Li, X.J., and Li, S. (2014). Age-dependent decrease in chaperone activity impairs MANF expression, leading to Purkinje cell degeneration in inducible SCA17 mice. *Neuron* 81(2), 349-365. doi: 10.1016/j.neuron.2013.12.002.

Supplementary Figure 1.

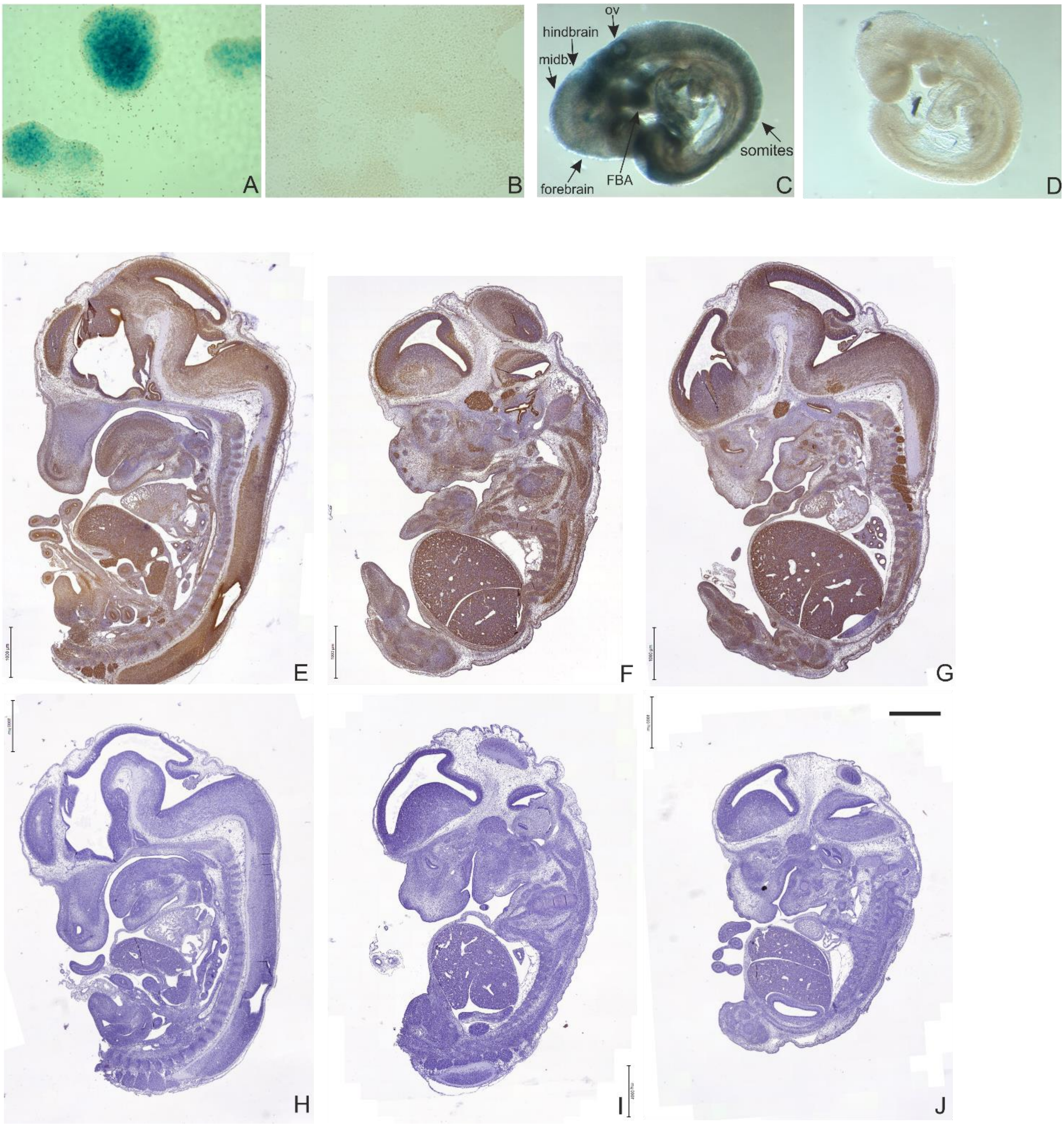

Supplementary Figure 1. LacZ staining and MANF immunohistochemistry on sections from mouse embryos at E13.5 mouse embryo

(A-B) X-gal staining in targeted *Manf*$^{+/-}$ embryonic stem cells representing MANF expression. No LacZ staining was observed in *Manf*$^{+/+}$ embryonic stem cells.

(C-D) LacZ staining in E9.5 *Manf*$^{+/-}$ mouse embryo representing MANF expression. No LacZ staining was observed in *Manf*$^{+/+}$ embryo at E9.5. midb. – midbrain, ov – otic vesicle, FBA - first branchial arch.

(E-J) Low magnification of the E13.5 mouse embryo stained with anti-MANF antibody, where *Manf* $^{+/+}$ **(E-J)** *and Manf* $^{-/-}$ **(H-J)**. Scale bar, 1000 µm.

Supplementary Figure 2.

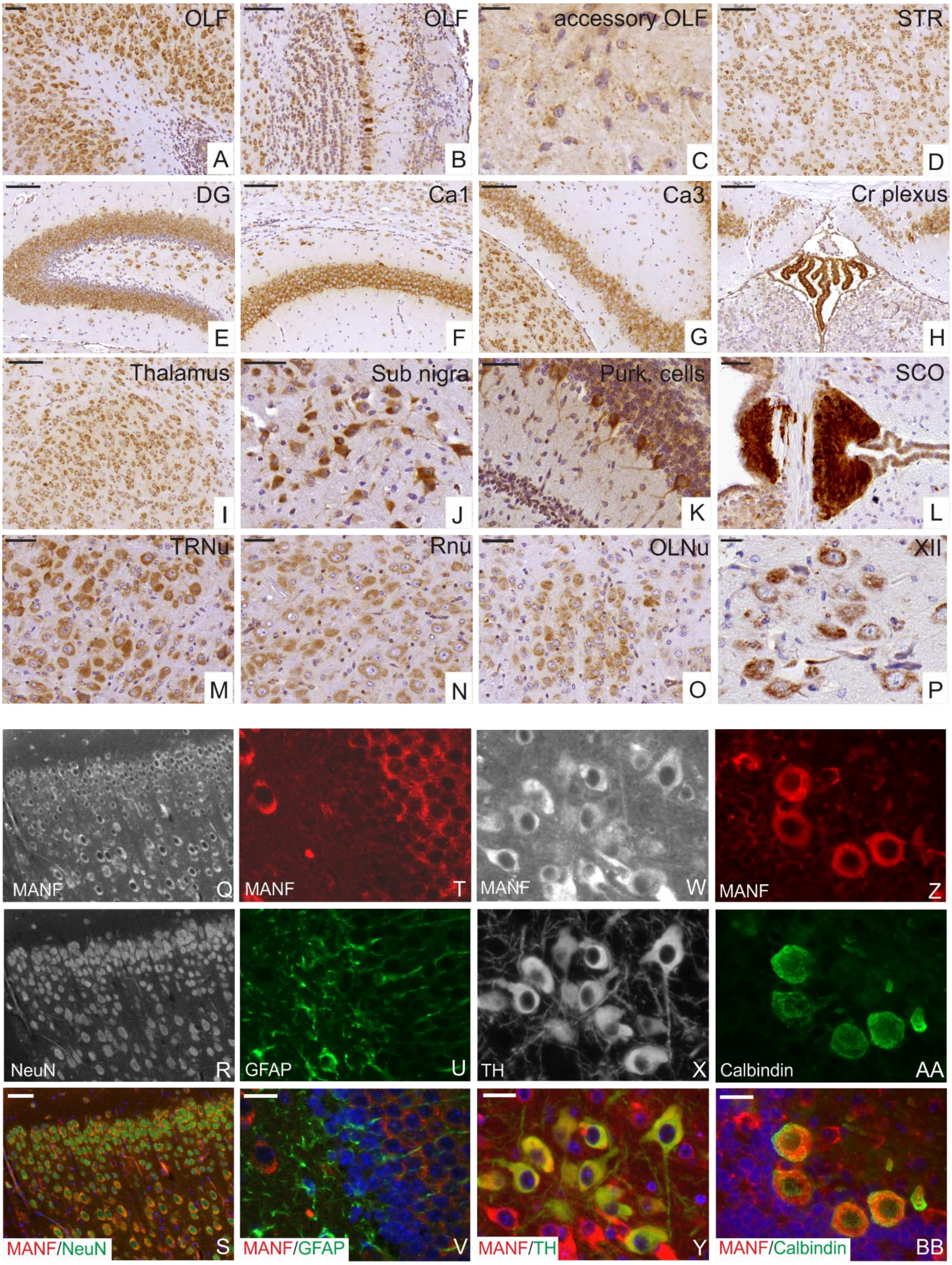

Supplementary Figure 2. MANF expression in mouse brain at postnatal day P14.

(A) Anterior olfactory bulb. Scale bar 50 µm. **(B)** Mitral cell layer of olfactory bulb. Scale bar 100 µm. **(C)** accessory olfactory bulb. Scale bar 20 µm. **(D)** Striatum (STR). Scale bar 100 µm.

(E-G) High levels of Manf expression observed in the pyramidal layers of hippocampus. **(E)** Dentate gyros (DG). Scale bar 100 µm. **(F)** CA1. Scale bar 100 µm. **(G)** CA3. Scale bar 100 µm.

(H) Strong expression of MANF in Choroid plexus (Cr plexus). Scale bar 100 µm.

(I) MANF signal in Thalamus. Scale bar 100 µm. **(J)** Substantia nigra (Sub nigra). Scale bar 50 µm.

(K) Purkinje cells (Purk cells). Scale bar 20 µm. **(L)** MANF immunohistochemistry in subcommissural organ (SCO). Scale bar 50 µm.

(M) Tegmental reticular nucleus (TRNu). Scale bar 50 µm. **(N)** Red nucleus (Rnu). Scale bar 50 µm. **(O)** Oculomotor nucleus (OLNu). Scale bar 50 µm. **(P)** Hypoglossal nucleus (XII). Scale bar 20 µm.

(Q-BB) Double immunohistochemistry analysis with anti-MANF antibody **(Q, T, W, Z)** and antibodies against other neuronal markers , neuron-specific protein (NeuN) **(R)**, Glial fibrillary acidic protein (GFAP) **(U),** Calbindin **(H)**,Tyrosine hydroxylase (TH) **(X),** Calbindin positive Purkinje cell **(AA)**. MANF (red) was co-expressed with NeuN positive neurons (green) in the mouse cortex **(S)**, but not co-localized with GFAP (green) positive astrocytes in mouse hippocampus **(V)**. MANF expression in TH positive neurons in substantia nigra **(X).** MANF (red) co-localized with Calbindin positive Purkinje cell (green) in cerebellum **(BB)**. **(Q-S)** Scale bar, 50 µm. **(T-BB)** Scale bar, 20 µm.

Supplementary Figure 3.

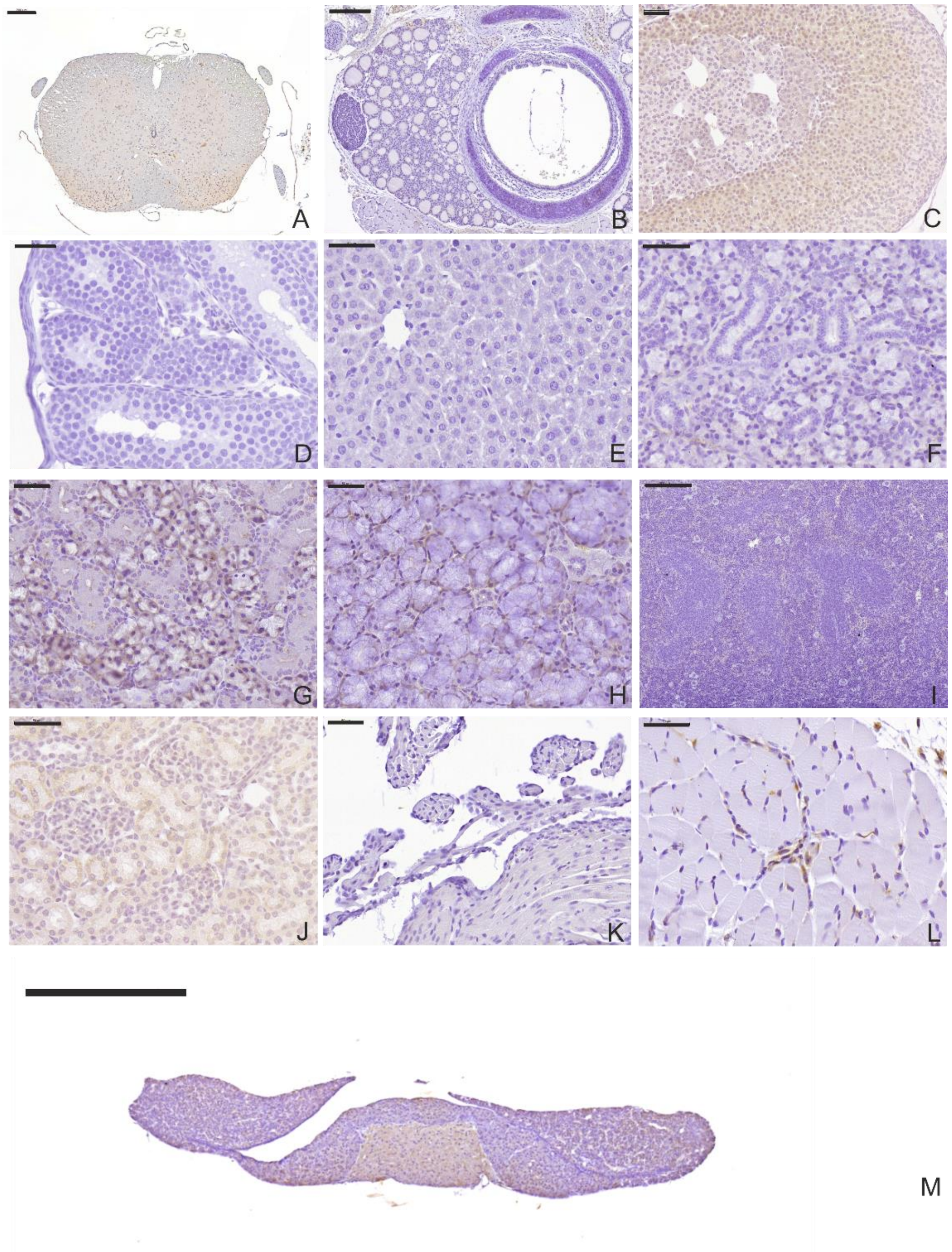

Supplementary Figure 3. ***Manf*$^{-/-}$ tissues stained with anti-MANF antibodies as a negative control**

(A-L) *Manf*$^{-/-}$ mouse tissues stained with anti-MANF antibody. **(A)** Spinal cord. Scale bar, 200 µm. **(B)** Thyroid gland, trachea. Scale bar, 200 µm. **(C)** Adrenal gland. Scale bar, 50 µm. **(D)** Testis. Scale bar, 50 µm. **(E)** Liver. Scale bar, 50 µm. **(F-H)** Sublingual gland **(F)**, submandibular gland **(G)** and parotid gland **(H)** of salivary gland. **(F-H)** Scale bar, 50 µm. **(I)** Spleen. Scale bar, 200 µm. **(J)**

Kidney. Scale bar, 50 µm. **(K)** Cardiac muscle. Scale bar, 50 µm. **(L)** Muscle. Scale bar, 50 µm. **(M)** Pituitary gland. Scale bar, 1000 µm.

Supplementary Figure 4.

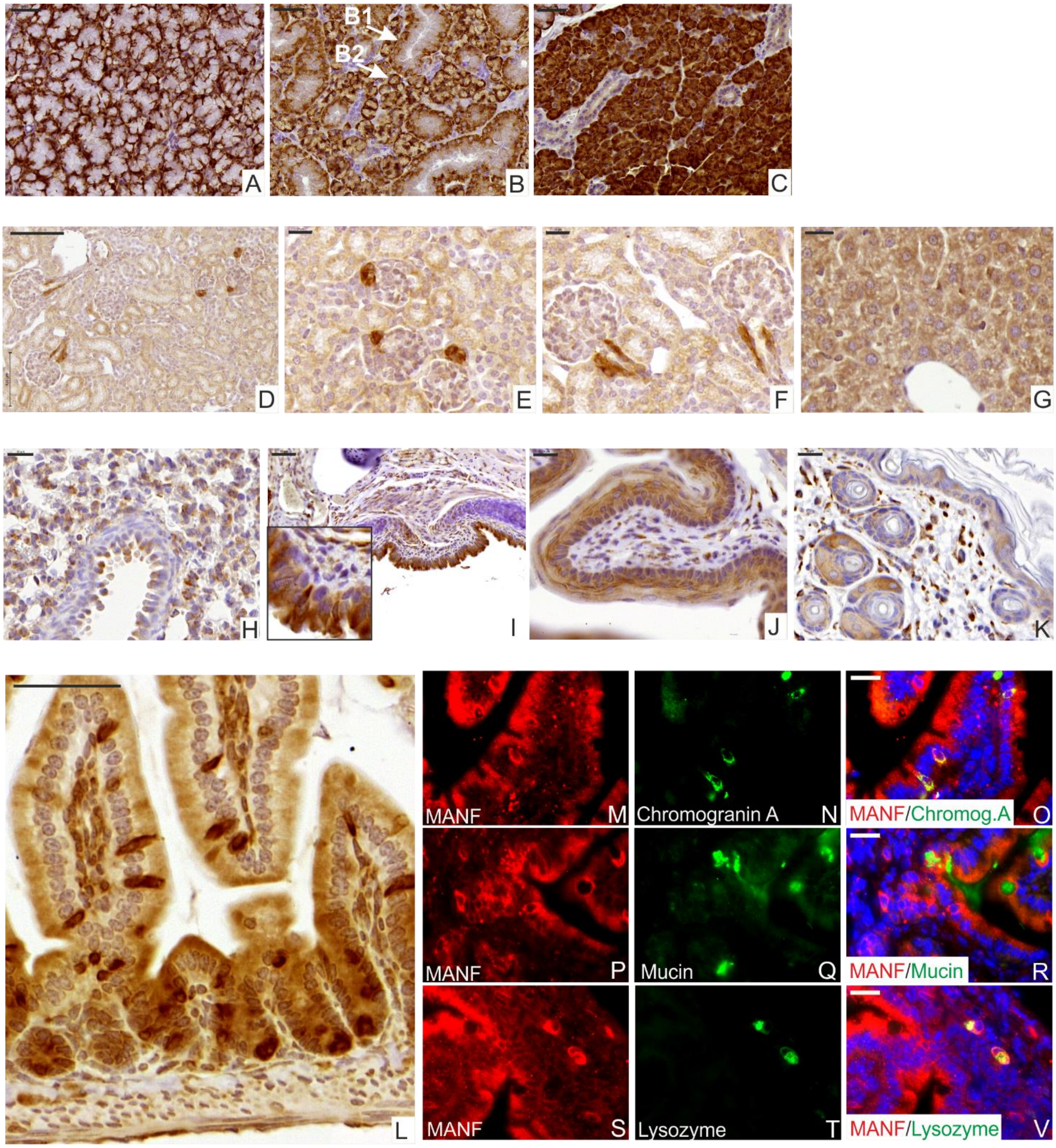

Supplementary Figure 4. MANF expression in other mouse tissues

(A-C) High levels of MANF expression was observed in different parts of mouse salivary gland at postnatal age P56. Scale bar, 50 µm. **(A)** MANF expression in the cells of sublingual gland. **(B)** MANF expression was detected in both mucous **(B1)** and serous acini **(B2)** of submandibular gland. **(C)** MANF expression in the secretory acini cells of parotid gland.

(D-F) Low levels of MANF expression was detected in the tubules and glomerulus of mouse kidney and moderate MANF expression was found in the cells of the juxtaglomerular apparatus at postnatal age P14. Scale bar, 20 µm.

(G) Moderate levels of MANF expression was observed in hepatocytes of mouse liver at postnatal age P14. Scale bar, 20 µm.

(H) Low levels of MANF expression in the mouse lungs. Scale bar, 20 µm. **(I)** Strong expression of MANF was observed in the tracheal epithelial cells at the postnatal age P14. Scale bar, 50 µm. **(J)** Moderate levels of MANF expression was detected in the epithelium of oesophagus at the postnatal age P14. Scale bar, 20 µm. **(K)** MANF expression was detected in the cell of dermis, sebaceous glands and in the epithelium cells of the mouse skin at P14. Scale bar, 20 µm.

(L) MANF expression in the duodenum of the mouse intestine at P14. Scale bar, 50 µm. **(M-V)** Double immunohistochemistry analysis with anti-MANF antibody **(M, P, Q)** and anti-chromogranin A **(N)**, anti-mucin **(Q)** and anti-lysozyme **(T)**. MANF (red) was co-expressed with chromogranin A, mucin and lysozyme (red) in the villi of P14 duodenum **(O, R, V).** Cell nuclei were labelled with DAPI (blue). Scale bar, 20 µm.

Supplementary Figure 5.

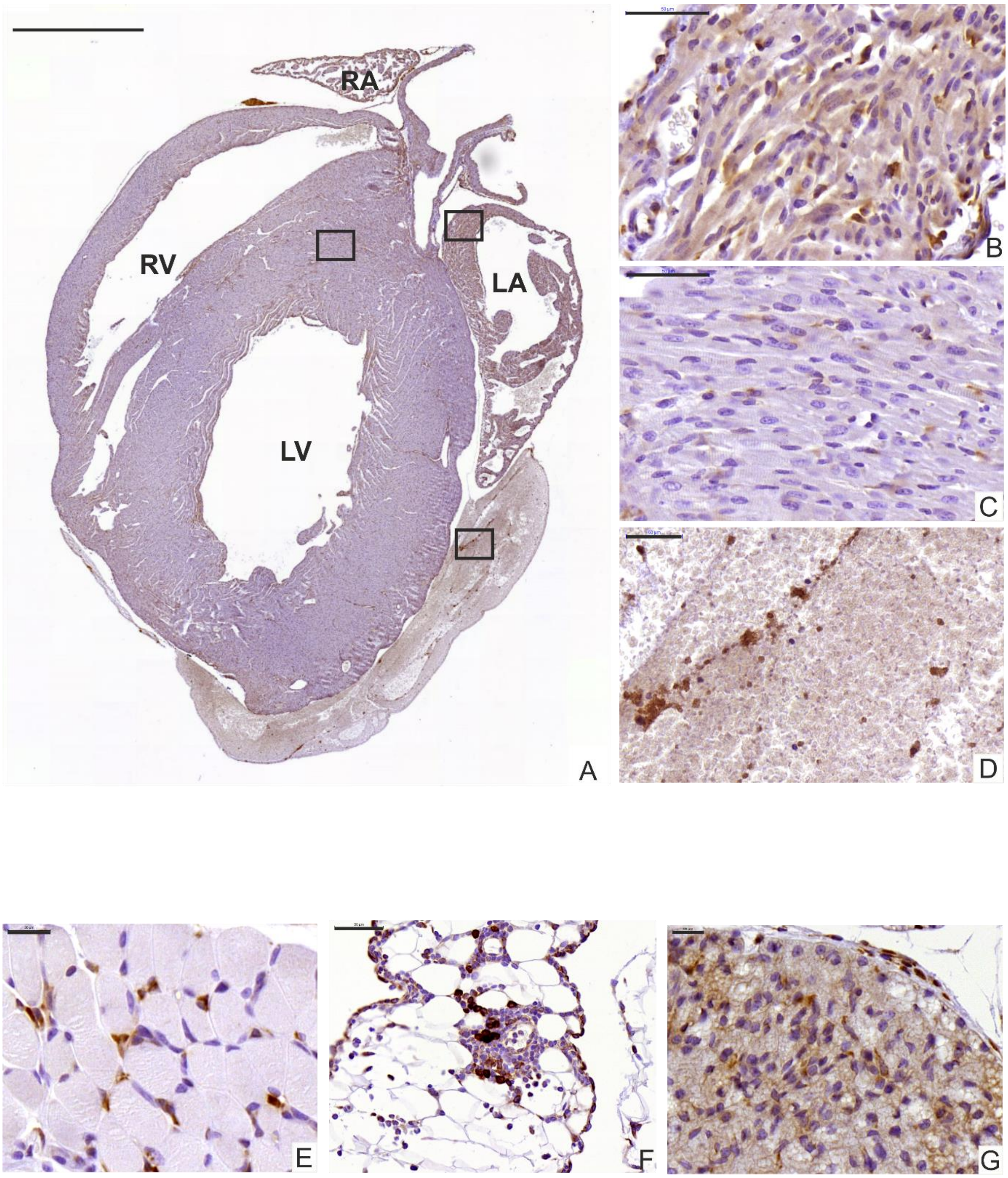

Supplementary Figure 5. MANF expression in mouse cardiac muscle, skeletal muscle and fat tissue

(A-D) Low levels of MANF expression was observed in the mouse heart. **(A)** Low magnification of the mouse heart at postnatal stage P14 stained with MANF antibody. RV - right ventricle, LV - left

ventricle, RA - right atrium, LA - left atrium. Scale bar, 1000 µm. **(B)** MANF positive cells in the cells of atrium tissue. Scale bar, 50 µm. **(C)** Only few MANF positive cells were observed in the cells of myocardium. Scale bar, 50 µm. **(D)** MANF expression was detected in several cell of the epicardial adipose tissue. Scale bar, 50 µm.

(E) Few MANF positive cells were noted in the mouse muscle. Scale bar, 20 µm.

(F-G) MANF expression was detected in white adipose tissues **(F)** and brown adipose tissue **(G)**. **(F)** Scale bar 50 µm. **(G)** Scale bar 20 µm.

Supplementary Figure 6.

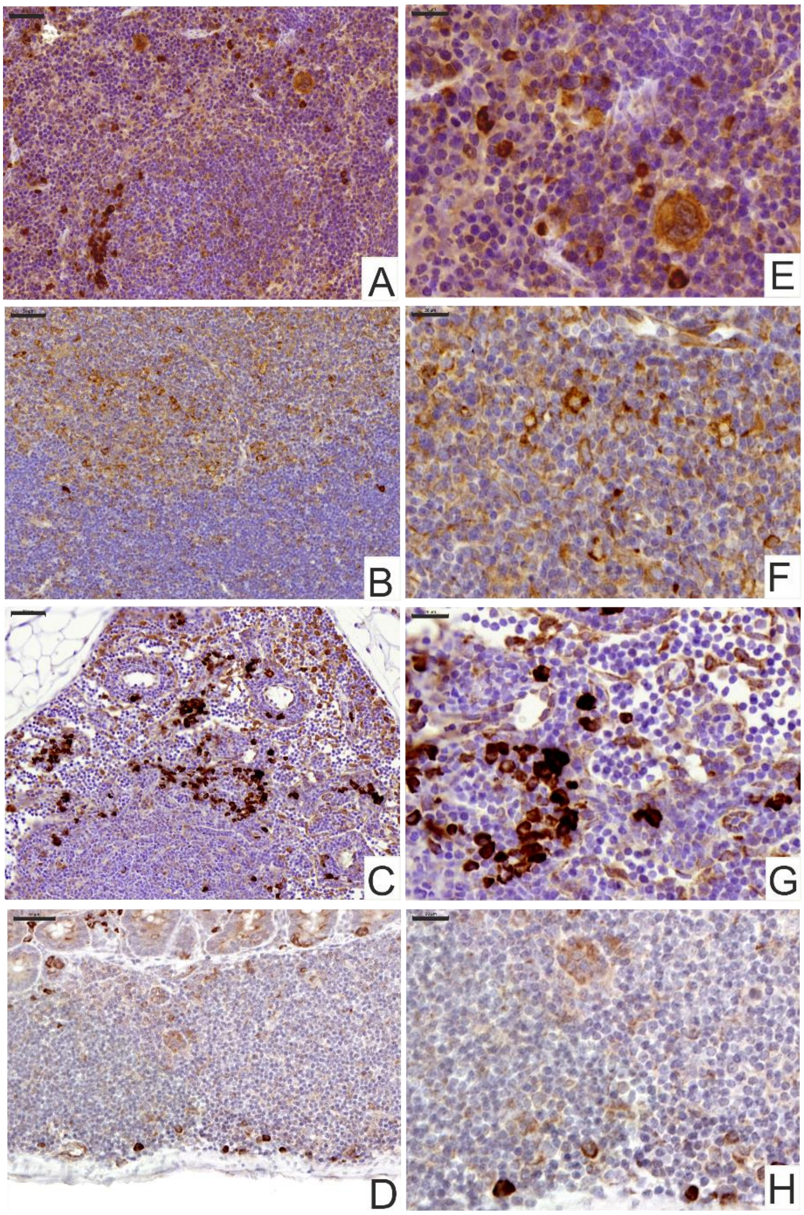

Supplementary Figure 6. MANF expression in the mouse lymphatic system.

MANF positive cells were found in the spleen **(A, E)**, thymus **(B, F)**, lymph node **(C, G)**, payer's patch **(D, H)**. **(A-D)** Scale bar, 50 µm. **(E-H)** Scale bar, 20 µm.

Supplementary Figure 7.

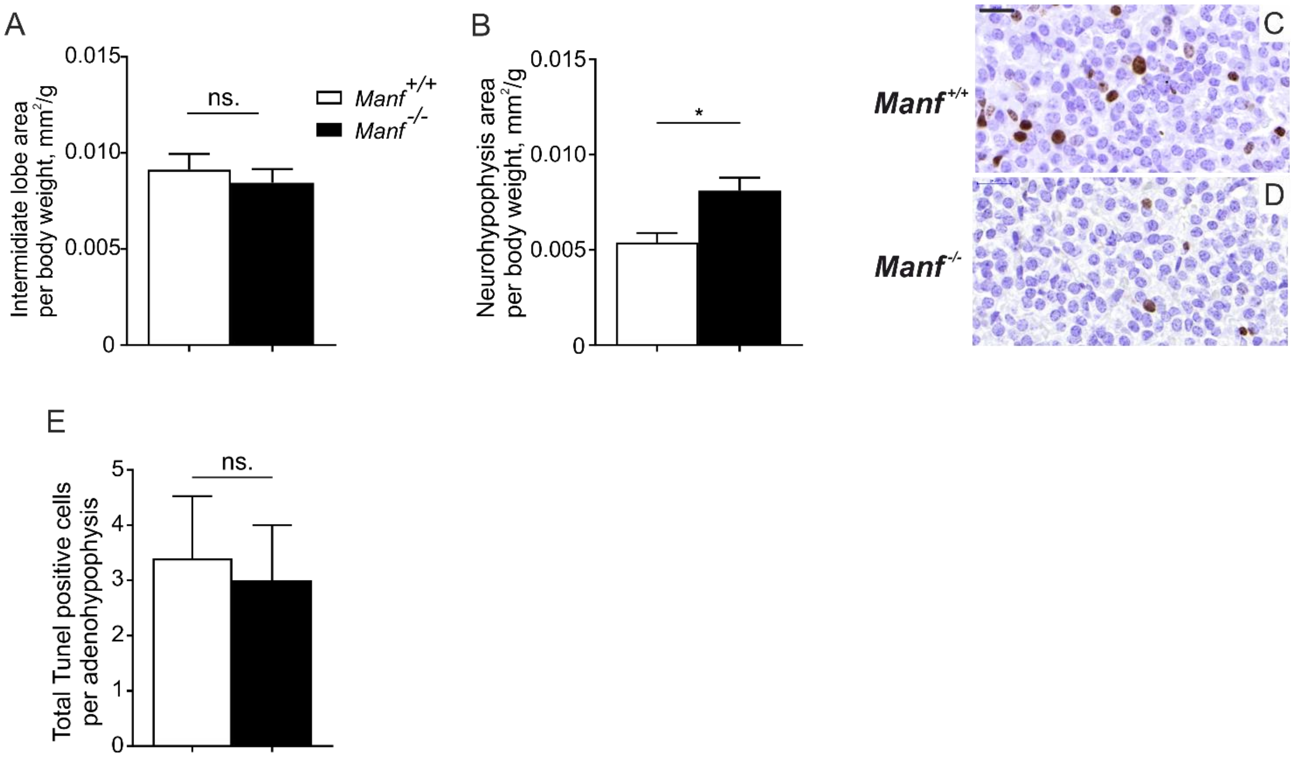

Supplementary Figure 7. Ablation of MANF in the conventional *Manf*$^{-/-}$ mice results in abnormal development of the adenohypophysis in the pituitary gland.

(**A**) Area of intermediate lobe normalized to body weight of the animal (mm^2/g).

(**B**) Area of neurohypophysis normalized to body weight of the animal (mm^2/g).

(C-D) Ki67 immunohistochemistry on adenohypophysis of pituitary glands from *Manf* $^{+/+}$ **(C)** and *Manf* $^{-/-}$ **(D)** mice at postnatal stage P42. Scale bar, 20 µm.

(E) Apoptosis of adenohypophyses assessed by Tunel staining, *n* =3 mice per group.

Supplementary Tables

Supplementary Table S1. Dynamic range of the mouse CDNF ELISA. For each standard point, the mean accuracy and precision values of six runs are shown. The mean accuracy and precision were within ±10% RE and 10% CV, respectively. The individual accuracy values were within ±15% RE of the nominal values, and precision values were within 15% CV.

**Recombinant mouse CDNF (pg/ml)**	**15.6**	**31.3**	**62.5**	**125.0**	**250.0**	**500.0**
Mean recovery	14.74	30.80	66.57	134.78	260.41	453.91
Mean % RE	94.4	98.6	106.5	107.8	104.2	90.8
Range % RE (min–max)	85.6–109.9	86.9–112.8	102.4–110.4	93.6–111.3	101.2–106.2	86.4–101.4
Mean % CV	6.9	4.9	2.7	2.5	2.6	4.8
Max % CV	11.3	9.3	5.5	5.9	5.8	11.8

RE = relative error, CV = coefficient of variation

Supplementary Table S2. Intra-assay precision (repeatability) and inter-assay precision (reproducibility) of the mouse CDNF ELISA. Results are shown as a mean of ten replicates for the intra-assay precision, and as a mean of six separate assays for the inter-assay precision, respectively.

**Level of added**	**Intra-assay precision**		**Inter-assay precision**	
**rec. mouse CDNF**	Mean (pg/ml)	% CV	Mean (pg/ml)	% CV
Low	31.2	12.1	20.3	7.5
Medium	188.6	9.7	131.6	8.7
High	409.5	9.0	424.0	4.2
	**Average**	**10.3**	**Average**	**6.8**

CV = coefficient of variation

Supplementary Table S3. Specificity of the mouse CDNF ELISA. The assay gave values below the ELISA sensitivity level (6 pg/ml) for tissue samples from CDNF KO mouse. In contrast, the assay gave high values to the parallel samples from WT mouse prepared and diluted identically to the KO mouse samples.

**Sample**	**Abs**	**pg/ml**
WT salivary gland	3.372	963.8
KO salivary gland	-0.005	n.d.
WT brain	2.620	875.9
KO brain	0.015	1.2
WT kidney	3.120	875.9
KO kidney	0.017	1.4

WT = wild type, KO = knock out (*Cdnf* $^{-/-}$), Abs = absorbance at 450nm (minus 540nm and mean blank absorbance value), n.d., not detected.

Supplementary Table S4. Linearity of dilution (%) of endogenous CDNF in mouse tissue lysates. The recovery in 2nd and 3rd dilution was calculated compared to the measured concentration in the 1st dilution in the dilution series.

	**Measured concentration (pg/ml)**			**x Dilution factor (pg/ml)**			**Recovery (%)**	
**Dilution**	**1st**	**2nd**	**3rd**	**1st**	**2nd**	**3rd**	**2nd**	**3rd**
WT brain (1:10, 1:20, 1:40)	131.1	69.8	34.6	1 311	1 396	1 385	106	106
WT liver (1:5, 1:10, 1:20)	100.8	48.6	23.6	504	486	472	96	94
WT pancreas (1:5, 1:10, 1:50)	182.5	87.2	19.0	913	872	951	96	104
WT pituitary (1:5, 1:10, 1:50)	290.6	150.1	27.5	1 453	1 501	1 377	103	95
WT olf. bulb (1:5, 1:10, 1:50)	182.7	91.9	15.7	914	919	784	101	86
					**Mean recovery (%)**		**100**	**97**

WT = wild type; olf. olfactory.

Supplementary Table S5. Recovery (%) of spiked recombinant mouse CDNF in wild type and CDNF KO mouse tissue lysates.

	**Measured concentration (pg/ml)**			**Minus endogenous CDNF**		**Recovery (%)**	
**Spike (pg/ml)**	**-**	**100**	**250**	**100**	**250**	**100**	**250**
KO brain (1:4)	1.6	115.1	230.7	113.5	229.1	114	92
WT brain (1:4)	285.9	391.4	497.8	105.5	211.9	105	85
WT pancreas (1:10)	116.1	251.0	406.9	134.8	290.7	135	116
					**Average**	**118**	**98**

WT = wild type, KO = knock out (*Cdnf*$^{-/-}$)
